# Expanding the range of the respiratory infectome in Australian feedlot cattle with and without respiratory disease using metatranscriptomics

**DOI:** 10.1186/s40168-023-01591-1

**Published:** 2023-07-25

**Authors:** Barbara P. Brito, Melinda J. Frost, Kay Anantanawat, Frederick Jaya, Tony Batterham, Steven P. Djordjevic, Wei-Shan Chang, Edward C. Holmes, Aaron E. Darling, Peter D. Kirkland

**Affiliations:** 1 Australian Institute for Microbiology & Infection, University of Technology Sydney, Ultimo, New South Wales Australia; 2 New South Wales Department of Primary Industries, Elizabeth Macarthur Agricultural Institute, Menangle, New South Wales Australia; 3 Present Address: Biosecurity and Food Safety, NSW Department of Primary Industries, Elizabeth Macarthur Agricultural Institute (EMAI), Menangle, New South Wales Australia; 4 Illumina Australia, Ultimo, New South Wales Australia; 5 Apiam Animal Health, East Bendigo, Australia; 6 Sydney Institute for Infectious Diseases, School of Medical Sciences, The University of Sydney, Sydney, NSW Australia

**Keywords:** Bovine respiratory disease, Metatranscriptomics, Metagenomics, Infectome, Virome, Microbiome

## Abstract

**Background:**

Bovine respiratory disease (BRD) is one of the most common diseases in intensively managed cattle, often resulting in high morbidity and mortality. Although several pathogens have been isolated and extensively studied, the complete infectome of the respiratory complex consists of a more extensive range unrecognised species. Here, we used total RNA sequencing (i.e., metatranscriptomics) of nasal and nasopharyngeal swabs collected from animals with and without BRD from two cattle feedlots in Australia.

**Results:**

A high abundance of bovine nidovirus, influenza D, bovine rhinitis A and bovine coronavirus was found in the samples. Additionally, we obtained the complete or near-complete genome of bovine rhinitis B, enterovirus E1, bovine viral diarrhea virus (sub-genotypes 1a and 1c) and bovine respiratory syncytial virus, and partial sequences of other viruses. A new species of paramyxovirus was also identified. Overall, the most abundant RNA virus, was the bovine nidovirus. Characterisation of bacterial species from the transcriptome revealed a high abundance and diversity of *Mollicutes* in BRD cases and unaffected control animals. Of the non-Mollicutes species, *Histophilus somni* was detected, whereas there was a low abundance of *Mannheimia haemolytica*.

**Conclusion:**

This study highlights the use of untargeted sequencing approaches to study the unrecognised range of microorganisms present in healthy or diseased animals and the need to study previously uncultured viral species that may have an important role in cattle respiratory disease.

Video Abstract

**Supplementary Information:**

The online version contains supplementary material available at 10.1186/s40168-023-01591-1.

## Background

Respiratory disease is one of the most common causes of disease and death in intensively managed cattle. While the severity of bovine respiratory disease (BRD) varies, it can account for up to 70% of the mortality observed in intensive beef production systems (i.e., feedlot cattle) [1]. Mild or severe clinical BRD affects an estimated 17% of animals within the first weeks of arrival into the feedlot [2]. Despite being one of the most studied diseases in livestock, the impact of BRD on the industry remains high. Bovine respiratory disease has a complex aetiology that includes a combination of stressors, individual host factors and a varying number of pathogens.

While some viruses and bacteria have been widely studied and are known to be an important component of the respiratory complex, novel, emerging or unrecognised pathogens are not easily detected because they are not routinely considered in the differential diagnosis or laboratory diagnostic assays are not available. Metagenomics approaches have facilitated the detection of complete microbiomes and viromes, including emerging pathogens or pathogens that cannot be easily cultured and isolated. Untargeted RNA sequencing (i.e., metatranscriptomics) can help detect the complete taxonomic profile of eukaryotic organisms, bacteria, or DNA viruses, as well as the genomic RNA of RNA viruses, enabling the identification of viral species in a single assay [3–6]. A few studies have used ‘shot-gun’ metagenomics approaches to characterise the respiratory virome of cattle [7–9]. Importantly, not only did these studies identify previously unrecognised viral species, but they also consistently observed an abundance of viruses not part of the regular veterinary diagnostic laboratory detection range, including influenza D virus (IDV), bovine rhinitis viruses A and B (BRAV and BRBV), parvoviruses and bovine herpesviruses other than bovine alphaherpesvirus-1 (BoHV-1).

Bacterial species are also key components of BRD. *Histophilus somni*, *Mycoplasma bovis*, *Pasteurella multocida* and *Mannheimia haemolytica* are often associated with disease [10]. Unfortunately, the use of antibiotics across different livestock systems, as well as its use in humans, has resulted in a widespread emergence of antimicrobial resistance (AMR), an increasing global public health threat. The resistance of BRD associated pathogens in cattle to treatment have been reported, but studies are in their infancy [11–14]. The use of metagenomics and metatranscriptomics integrated with phenotypic data can further our understanding of AMR acquisition and spread through the characterisation of a bacterial functional profile, including the expression of AMR genes.

Besides its critical application in understanding complex diseases, characterising the microbiome and virome of livestock in different geographic locations is a requisite for producing a global catalogue of microbial genomes. While the SARS-CoV-2 pandemic revealed the importance of sequencing viruses from wildlife species, it is also essential to determine the virome of agricultural production animal species due to their close contact and interaction with humans [15]. The scarcity of genomic data of pathogens from domesticated species highlights the need to foster global efforts to characterize the complete assemblage of viruses and other pathogenic organisms. With respect to viruses, there is a particular need to generate a robust reference database that allows future automation bioinformatics data analyses for metagenomics and, in turn, incorporate this tool in veterinary and public health laboratories for monitoring and surveillance of viral pathogens.

Most studies of BRD have been hampered by a dependence on a priori knowledge and hence have focused on the role of a limited number of pathogens and use of associated diagnostic tests [16]. In this study, we characterise the infectome of feedlot cattle with and without clinical BRD. Specifically, we define the infectome as the collection of bacteria, viruses, parasites, and fungi identified by deep shotgun RNA sequencing.

## Methods

### Sample collection and preparation

#### BRD clinical cases and non-affected ‘control’

Samples were collected from two Australian feedlots located in the state of New South Wales in April and November 2019. Both feedlots vaccinate animals intranasally with a BoHV-1 modified live vaccine (Rhinogard, Zoetis), and an inactivated vaccine against *Mannheimia haemolytica* and BoHV-1 (Bovilis MH + IBR, Coopers), administered intramuscularly. Vaccines were administered at feedlot arrival. At the feedlot, animals with signs of respiratory disease (rhinorrhea, cough, laboured breathing, nasal and ocular discharge, lethargy, or fever) were separated from normal cattle in the pens by experienced stockmen and confirmed by the veterinarian collecting the samples. Cases were defined as animals diagnosed with clinical respiratory disease with no prior treatment. Most animals included in this study were sampled before 40 days of arrival in the feedlot. For comparison, samples from animals without respiratory disease were either taken from healthy animals before being transported to the abattoir or from animals being evaluated for a condition other than BRD within 2 days of BRD cases collected. In this study, we refer to this comparison group of animals that were not clinically affected with BRD at the time of sampling as ‘controls’. The cattle sampling procedure was approved by the University of Technology Sydney Animal Care and Ethics Committee #ETH19-3407.

#### Sampling method

Animals were restrained in a crush for sampling. Nasal and deep nasopharyngeal swabs were collected from all animals. For nasal swabs, sterile 80 mm FLOQSwabs (Copan®) were introduced into the nasal cavity and rotated against the nasal epithelium. For deep pharyngeal swab collection, double guarded culture swabs (Har-Vet™) were used. The distance between the nostril and medial canthus of the eye was measured and used as the distance that the swabs were introduced into the ventral meatus. The swab tip was pushed out of the protecting guard, rotated against the pharyngeal mucosa for 5–10 s and retracted into the guard before removal from the nose. During the sample collection, the nasopharyngeal was collected first, followed by the nasal swabs. Both swabs were collected from both cavities.

Samples were collected from both feedlots in April 2019 and additional samples from one feedlot in November 2019. For the April 2019 collection, swabs from three animals of the same category (case/unaffected controls) were pooled (Additional File 1) into one sterile 10 mL tube containing transport medium (eNAT, Copan®) and Universal Transport Medium (Copan®). For the November 2019 collection, swabs were individually deposited into sterile 5 mL single tubes without transport medium, and immediately snap-frozen in dry ice. All sample tubes were transported on dry ice to the Bioscience Laboratory at the University of Technology Sydney for RNA extraction. RNA in samples collected without transport media was extracted within 3 weeks and within 2 months in samples collected with transport media.

### RNA extraction, sequence library preparation, and RNA sequencing

RNA extraction was undertaken using the RNeasy Plus Micro Kit and RNeasy Plus Mini Kit (Qiagen). For each sample collected, the concentration of purified RNA was measured using the Qubit™ RNA HS Assay Kit (Thermofisher). RNA was pooled to reach a concentration of 2–4 μg of RNA. A total of 10 cases and 5 control RNA sequencing libraries were prepared. All RNA sequencing libraries included samples from both nasal and nasopharyngeal swabs. The RNA quality of the pools was measured using a Bioanalyzer instrument (Agilent). Library preparation was completed using TruSeq Stranded Total RNA Human/Mouse/Rat Kit (Illumina) that removes cytoplasmic rRNA and sequenced in an Illumina NovaSeq S1 platform (paired-end sequencing with 150 cycles per read) at the Australian Genome Research Facility (AGRF).

### Read assembly

Quality trimming of the reads of the 15 RNA sequencing libraries was performed using BBDuk from BBTools 38.87 [17]. The reads were mapped to the *Bos taurus* genome (accession: GCA_002263795.2) using BWA-MEM and quantified using Samtools idxstats (Samtools 1.12) to determine the proportion of sequences that belonged to the host [18]. Viral genomes were assembled using SPAdes genome assembler (metaspades.py with options –only-assembler -k 21,31,41,51,61,71,81,91,101) v3.13.0 and MEGAHIT v1.2.9 (options –k-min 21 –k-max 141 –k-step 10) using quality trimmed reads with no prior removal of *Bos taurus*-mapped sequences [19, 20].

### Virus taxonomic classification

Taxonomic classification of contigs was performed using BLASTn 2.10.1 + to query the NCBI nucleotide database and diamond v2.0.4.142 (BLASTx) using the non-redundant protein database [21, 22]. All contigs classified as being derived from vertebrate host viruses were analysed downstream.

### Viral abundance determined via metatranscriptomics

To estimate viral abundance, we created a multi-fasta file with reference viruses and consensus viral genomes assembled in the previous step. When two genotypes of the same viral species were obtained, both were included in the reference file. Additionally, we included the reference genomes from bovine DNA viruses BoHV-1 (accession number MG407776.1), bovine gammaherpesvirus-6 (accession number KJ705001.1) and bovine alphaherpesvirus-5 (accession number KY559403.1). The quality trimmed reads were mapped to the indexed multi-fasta file using RSEM with the Bowtie2 aligner [23]. The abundance of viruses was expressed by the transcript per million normalisation (TPM). For segmented viruses (i.e. IDV), each segment was considered a transcript for the estimation of abundance. To display the overall abundance of segmented viruses, we estimated the mean TPM from all segments. Figures were created using R with the packages “tidyverse”, “ggplot2” and “cowplot” [24].

### Viral abundance determined via qRT-PCR

To estimate the relative viral concentration in case and comparison samples (*n* = 39), we performed qRT-PCR assays on samples collected in viral transport medium (each containing 3 swabs). Total nucleic acid was extracted from 50 μL of the sample using the MagMax 96 viral RNA kit (Thermofisher), run on a magnetic particle handling system (KF96, Thermofisher) as described previously [25]. qRT-PCR assays were used to detect BoNV, BCoV, IDV, BRAV and BRBV, novel bovine narmovirus-1, ON861830) and qPCR for DNA virus BoHV-1 (primers available in Additional File 2). Each qPCR/qRT-PCR assay used 5 μL of nucleic acid added to 20 μL of AgPath Mastermix (Thermofisher) and was run on a Quant-studio 5 Thermocycler (Thermofisher) for 45 cycles under the standard cycling parameters recommended for the master mix. The threshold was set at 0.05, and results were expressed as cycle threshold (Ct) values.

### Comparison of viral RNA detection in nasal swabs and nasopharyngeal swabs

To determine if the detection of a specific virus was associated with the sample collection site (nasal swab or deep nasopharyngeal swab), we compared the PCR results obtained from both specimens. The PCR results obtained for eight different viruses, as described above, were classified as positive where Ct ≤ 45 and negative for Ct > 45. The agreement for detection of each virus in nasal and nasopharyngeal swabs was determined using Cohen’s kappa [26].

### Association of selected viruses with bovine respiratory disease using qRT-PCR results

A logistic regression was performed to assess the association of viruses that had been identified in samples using qPCR (BoNV, BCoV, IDV, BRAV and BRBV, bovine narmovirus-1, BoHV-1) and the clinical BRD. The dependent variable was the disease status (BRD case or unaffected “control”), and explanatory variables were the Ct values for each of the viruses analysed in the samples and feedlot. Real-time PCR results were available for the nasal and nasopharyngeal swabs; only the swab result (nasal or nasopharyngeal) with the lowest Ct value for each virus was used in the regression. Backwards elimination was used to retain only variables with *p* < 0.05. All two-way interactions were included in the regression and tested for the variables with *p* < 0.1. The analyses were performed in R.; the regression was performed using the glm function in the stats package and plots were done using ggplot [24].

### Phylogenetic analyses

For selected viruses where the evolutionary relationship to global genotypes was relevant, we performed a phylogenetic analysis. Consensus assembled virus and representative global references collated from NCBI/GenBank sequences were aligned using MUSCLE and visualised in Aliview [27]. Maximum likelihood trees were estimated using IQ-TREE v1.6.7, incorporating the best-fit nucleotide substitution models (-m TEST) in each case and ultrafast bootstrap (-bb 1000) [28]. In the case of highly divergent viruses, an equivalent analysis was performed using amino acid alignments and their best-fit substitution models. The phylogenies were visualised and midpoint rooted using FigTree v1.4.4 [29]. Viruses were annotated using GATU and SnapGene® [30].

### Bacterial transcriptomics taxonomic classification, resistance profile and abundance

The taxonomic classification and respective abundance of the bacterial species in each of the pools were estimated using MetaPhlAn3.0 on the trimmed reads [31]. Relative abundance plots were generated across taxonomic ranks. We excluded unassigned reads and taxa with a relative abundance less than 1e − 5. We computed the alpha and beta diversity of metagenomes to determine if bacterial communities differed between case and control samples [32].

Relative abundance estimates from MetaPhlAn3.0 were multiplied by a constant of 1e + 6 and rounded to the nearest integer to obtain pseudo-counts to infer the within-host diversity across samples (alpha diversity). We calculated the observed Shannon, Simpsons and Fisher diversity using the pseudo-counts. The Kruskal–Wallis test was conducted for all four distances to determine if bacterial communities were significantly different between cases and unaffected animals.

The between-sample diversity (beta diversity) was calculated using Bray–Curtis dissimilarity. A non-metric multidimensional scaling (NDMS) plot was constructed using the distance matrix and the differences of beta diversities between cases and controls computed with PERMANOVA using the vegan v.2.5–7 package in R [33]. Filtering and plotting, as well as alpha and beta diversities, were conducted using phyloseq v1.34 [34].

Detection of antimicrobial resistant (AMR) genes was done using ABRicate on the metatranscriptome assembled contigs (https://github.com/tseemann/abricate) querying the Resfinder database [35]. Only AMR genes with > 90% nucleotide identity and 90% reference coverage were considered. To avoid including any AMR genes that may be present in the reagents or media, we queried the contigs where antimicrobial resistance genes were identified using NCBI VecScreen tool (https://www.ncbi.nlm.nih.gov/tools/vecscreen/) to detect potential vector contamination. Any strong match to a vector database of a contig containing an AMR gene was considered a contamination.

We estimated the AMR gene abundance by mapping the transcriptome back to the AMR sequences identified using RSEM [23].

### Eukaryotic classification

To determine the presence (gene expression) of eukaryotic bovine pathogens, we removed all reads that mapped to the host, bacteria and viruses using BWA-MEM and Samtools [18]. The remaining reads were taxonomically classified using CCMetagen [36]. We obtained the results as reads per million (RPM) and filtered out the hits that had < 1 RPM and less than 90% similarity to a known reference. We report fungi and nematodes that may be of pathogenic importance or known to infect cattle. The metric used was the reads per million (RPM) found in each of the libraries at the family, genus or species level. Significant differences between the abundance of relevant eukaryotic pathogens identified in cases and unaffected “control” libraries were assessed using a Mann–Whitney *U* test.

## Results

### Overview of sequenced libraries

In total, we generated 15 RNA sequencing libraries, comprising 10 from cases (L1–L10) and 5 from unaffected (L11–L15), representing 73 animals with BRD and 40 unaffected animals (Additional file 1). Libraries had a depth of 13–28 Gbp. The proportion of reads mapped to the *Bos taurus* genome ranged between 63.1 and 99.2% (median 83.1%). The sequenced metatranscriptomes were deposited at NCBI BioSample accession numbers 24043620–24043634.

### Assembled viral species

Complete or near-complete genomes of eight animal viral species (Virus name) were obtained: *bovine nidovirus 1* (bovine nidovirus; BoNV), *betacoronavirus 1* (bovine coronavirus; BCoV), *bovine rhinitis A virus* (BRAV), *bovine rhinitis B virus* (BRBV), *enterovirus E1* (enterovirus E1; bovine enterovirus; EV-E1), *pestivirus A* (bovine viral diarrhea virus-1; BVDV-1), *bovine orthopneumovirus* (bovine respiratory syncytial virus; BRSV), and *deltainfluenzavirus influenzae* (influenza D virus; IDV). Similarly, partial sequences of three viruses were obtained: *mamatrovirus* (bovine astrovirus; BAstro), *ungulate tetraparvovirus 1* (bovine hokovirus 1; BPARV4*)*, and *ungulate bocaparvovirus 6* (ungulate bocaparvovirus 6; UBPV6). These 12 varied in their nucleotide identity to published references: IDV, BCoV, BPARV4 and UBPV6 had nucleotide identities with > 95% similarity to published data, while BoNV, BVDV-1 and BAstro had identities with 90–95% similarity to published sequences. Picornaviruses BRAV and EV-E1, as well as BRSV, showed greater divergence from available published sequences (Table 1).

**Table 1:** Known viral species assembled from the RNA sequencing libraries. Twelve known RNA virus species and short DNA viruses were assembled. Reference library indicates the library from which a viral genome was obtained and used to estimate the nucleotide identity to the closest reference. Viruses with large DNA genomes (i.e. Herpesviruses) were not included due to the low coverage of the contigs obtained with RNA sequencing

Known viral sequences identified in samples	Consensus sequence RNA library	Reference Coverage	Nucleotide identity to closest reference	Closest reference accession number	Reference length	Assembly length	Assembly accession number
Bovine nidovirus (BoNV)-(a)	L6	100%	91.54%	KM589359.1	20261	20259	ON330458
Bovine nidovirus (BoNV)-(b)	L4	100%	91.39%	KM589359.1	20261	20331	ON330451
Bovine rhinitis A virus (BRAV)-3 (a)	L13	100%	83.2%	KT948520.1	7267	7367	OP020167
Bovine rhinitis A virus (BRAV)-3 (b)	L13	94%	83.10%	KT948520.1	7267	6799	OP020171
Bovine coronavirus (BCoV)	L3	100%	98.71%	FJ425189.1	30997	30983	OP020176
Enterovirus E1 (EV-E1)(a)	L12	99%	85.45%	MN598021.1	7447	7383	OP020148
Enterovirus E1 (EV-E1)(b)	L12	99%	85.29%	MN598021.1	7447	7386	OP020151
Influenza D virus (IDV) segment 1	L13	100%	99.13%	LC494105.1	2319	2319	OP020132
Influenza D virus (IDV) segment 2	L9	99%	98.23%	LC270266.1	2330	2314	OP020133
Influenza D virus (IDV) segment 3	L14	100%	98.45%	LC494107.1	2133	2183	OP020134
Influenza D virus (IDV) segment 4 (a)	L13	100%	99.25%	LC494108.1	1995	2033	OP020135
Influenza D virus (IDV) segment 4 (b)	L4	99%	96.4%	LC270268.1	2049	2028	OP020136
Influenza D virus (IDV) segment 5	L9	99%	98.36%	LC270269.1	1775	1766	OP020137
Influenza D virus (IDV) segment 6	L9	99%	98.67%	LC128434.1	1219	1203	OP020138
Influenza D virus (IDV) segment 7	L4	99%	97.68%	LC270271.1	868	862	OP020139
Bovine rhinitis B virus (BRBV)-5	L15	100%	79.52%	KU159360.1	7494	7506	OP020156
Bovine rhinitis B virus (BRBV)-2	L15	100%	88.68%	KU159357.1	7271	7505	OP020160
Bovine respiratory syncytial virus	L4	89%	87.24%	NC_038272.1	15140	13416	OP020146
Bovine viral diarrhea virus-1c	L6	100%	90.50%	M96751.1	12308	12287	OP020142
Bovine viral diarrhea virus-1a	L8	96%	91.44%	LR760748.1	12297	11788	OP020143
Bovine astrovirus (BAstro)	L15	70%	90.28%	KP264970.1	6099	1584/1745	OP020144-5
Bovine hokovirus 1 (BPARV4)^a^	L14	76%	99.55%	KU172423.1	5254	3969	OP020140
Ungulate bocaparvovirus 6(UBPV6)^a^	L6	25%	97.80%	KU172421.1	5224	1318	OP020141

^a^DNA viruses with small genomes

Three small contigs assembled (range 271–465 nucleotides) were classified as bovine rotaviruses coding for VP1, VP7 and NSP4 with amino acid identities between 71 and 100% to bovine and porcine rotavirus B and C (Additional file 3). Taxonomic classification of contigs also identified three bovine herpesvirus species: *bovine alphaherpesvirus 1* (BoHV-1), *bovine gammaherpesvirus 6* (BoHV-6) and bovine alphaherpesvirus-5 (BoHV-5).

In addition to recognised bovine viruses, we identified a novel paramyxovirus, provisionally named *bovine narmovirus 1* (Bulang virus, GenBank accession numbers: ON861830-3). The virus with the highest level of similarity (50.29%) obtained by NCBI BLAST protein query of the RNA-dependent RNA-polymerase (L-protein) was Mossman virus (NCBI reference accession: NP_958055.1) that belongs to the Narmovirus genus.

### Viral abundance

**Fig. 1:**
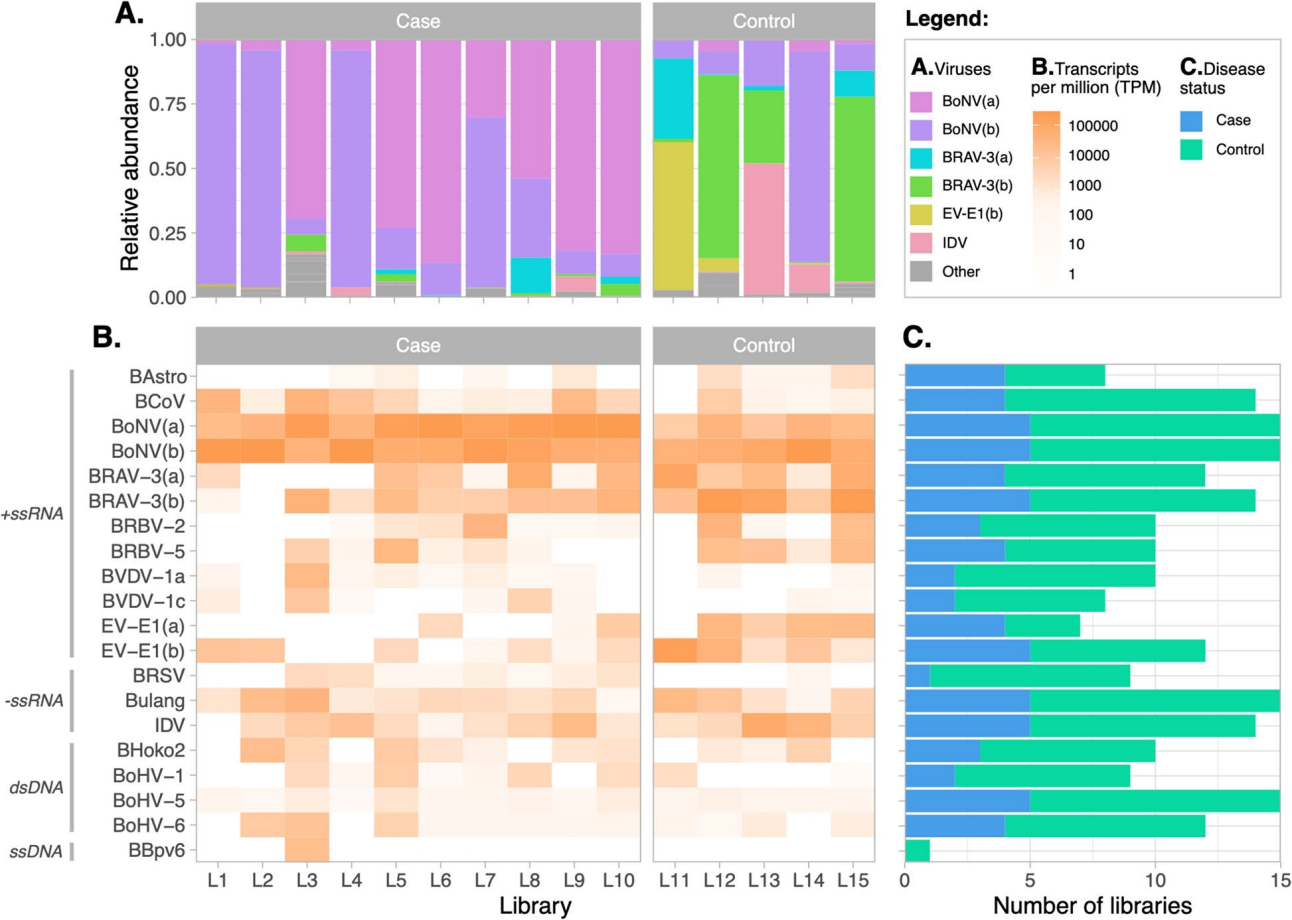
Composition, presence and abundance of viral species in all case (*n* = 10) and control libraries (*n* = 5). **A** Abundance of viral species in each of the libraries. **B** Heatmap of the transcripts per million (TPM) of each viral species per library. **C** Absolute number of case and control libraries where each viral species was present. BoNV genotypes (a) and (b), bovine narmovirus-1 and BoHV-5 were present in all libraries. BAstro, bovine astrovirus; BCoV, bovine coronavirus; BoNV, bovine nidovirus; BRAV, bovine rhinitis A virus; BRBV, bovine rhinitis B virus; BVDV, bovine viral diarrhea virus; EV-E1, enterovirus E1; BRSV, bovine respiratory syncytial virus; bovine narmovirus-1 (Bulang); IDV, influenza D virus; BPARV4, bovine hokovirus 1, BoHV, bovine herpesvirus; UBPV6, ungulate bocaparvovirus 6

Bovine nidovirus genotypes (a) and (b), bovine narmovirus-1 virus and BoHV-5 were present in all libraries (cases and unaffected control; Fig. 1). The abundance of BoNV genotypes (a) and (b) was the highest overall, being 4.2 and 13.4 times that of the second (BRAV) followed by enterovirus E-1 and influenza D virus (IDV). Bovine nidovirus had a high relative abundance in all case libraries, whereas bovine rhinitis A had a higher abundance in the libraries from unaffected animals.

To check for potential false positive identification of a viral species due to index hopping, we used RT-PCR to confirm the detection of a virus in any library with a low abundance which was sequenced in the same lane with a library that had a high abundance of that viral species. Only one virus, BRAV, had a potential false positive due to index hopping: Library 2 (L2) had a value of only 48 BRAV TPM, whereas L11 (run in the same instrument lane) had 262,214 BRAV TPM. BRAV RT-PCR for L2 was negative; therefore, we corrected the abundance of L2 to TPM = 0.

### Comparison of viral detection in nasal and nasopharyngeal swabs

**Fig. 2:**
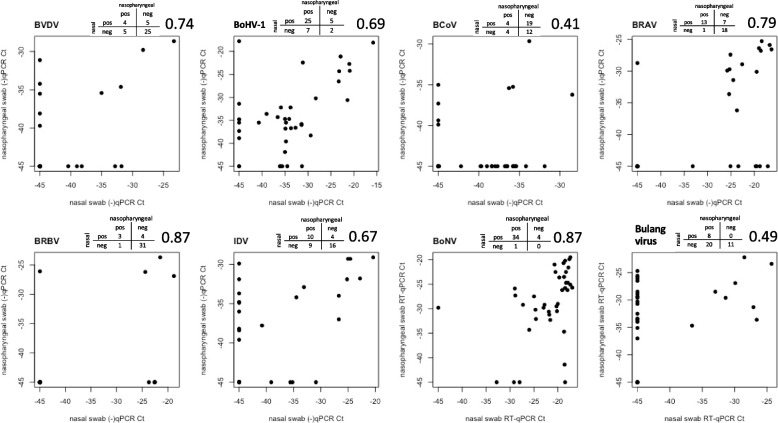
Scatterplot depicting the association of viruses detected by qPCR in nasal and nasopharyngeal swabs. The top area of each plot contains a 2 × 2 table with the number of swabs where a virus was detected (swabs were considered positive when the Ct value < 45) and the Cohen’s kappa percentage of agreement between the two sample types

We estimated the difference of specific virus detection within the nasal and deep nasopharyngeal swabs (Fig. 2). The lowest percentage agreement of the PCR values was 0.41 for BCoV where virus was detected more frequently in nasal swabs (23 positives) than nasopharyngeal swabs (8 positives). Bovine narmovirus-1 (Bulang virus) also had a low percentage of agreement (0.49), with 28 positive samples detected in nasopharyngeal swabs compared to only 8 positive nasal swabs. The remaining viruses identified with qPCR (BVDV, BoHV-1, BRAV, BRBV, IDV and BoNV) had a higher level of agreement (> 0.67) indicating a similar detection in both nasal and nasopharyngeal swabs.

### qRT-PCR of selected viruses

**Fig. 3:**
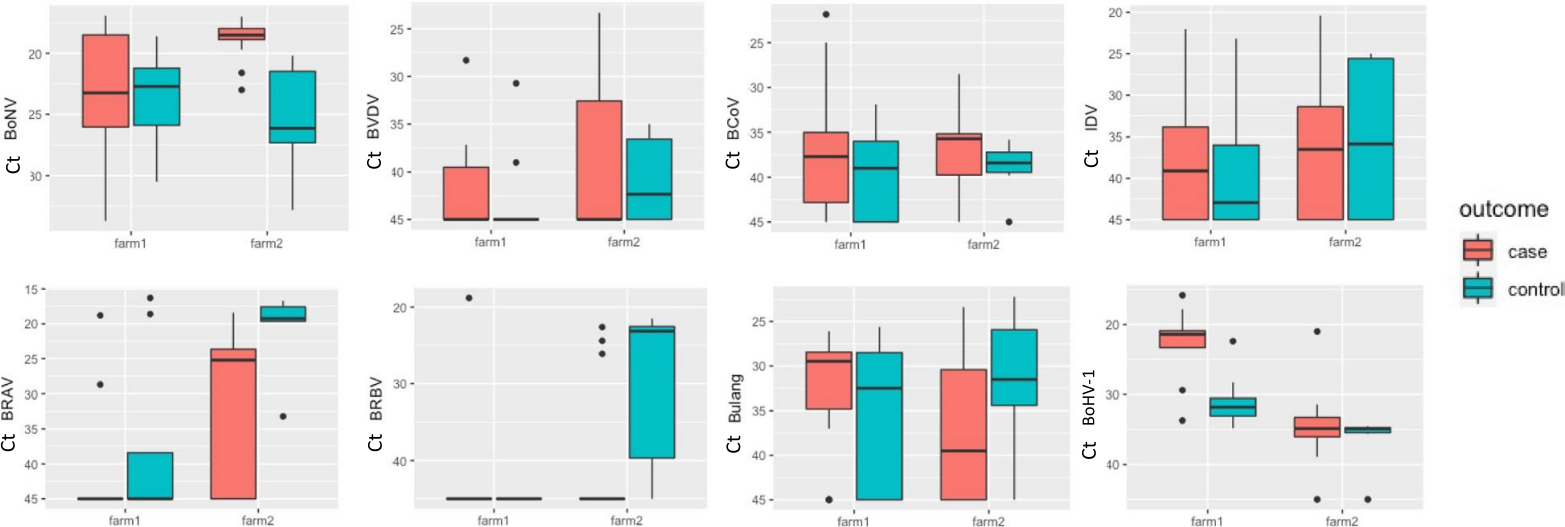
Boxplot of PCR results per feedlot and disease status (case or clinical bovine respiratory disease and BRD unaffected control). The *y*-axis are the Ct values (lower Ct values are a proxy to higher viral load). The colour of the boxplot represents the case or control status, and the *x*-axis for each virus plot group the results by feedlot (farm). The number of sample tubes represented are as follows: feedlot 1 cases *n* = 9; controls *n* = 8, feedlot 2 cases *n* = 16; controls = 6. In total, these sample tubes contained pooled swabs from 113 animals

Logistic regression analysis showed that two viruses were significantly associated with cases: BoNV (*p* = 0.03) and BoHV-1 (DNA virus; *p* = 0.02), whereas BRAV was significantly associated with BRD unaffected ‘control’ animals (*p* < 0.05). These results are consistent with the RNA-seq library abundance of BoNV and BRAV. There were no significant interaction terms. The feedlot had a significant effect on the logistic regression. We explored the feedlot effect by visualising the qPCR results with a boxplot (Fig. 3). At feedlot 1, there were significantly lower Ct values for BoHV-1 in cases compared to other animals, but this difference was not present in feedlot 2 (Fig. 3). Conversely, feedlot 2 cases had a significantly higher load of BoNV RNA compared to other animals, but this difference was not observed in feedlot 1. The virus load (as inferred by Ct values) for BRAV was lower in BRD cases at feedlot 2 (Fig. 3).

Of the 39 samples (pools of 3 animals) collected from cases and unaffected controls, BoNV was detected by PCR in 34 samples. The PCR Ct value was low (≤ 25) in 31 samples (Fig. 3). BVDV was detected in 14 samples (10/25 cases and 4/14 unaffected controls). BCoV was found in 18/25 cases and 9/14 controls, without an apparent association with BRD (Fig. 3). With respect to IDV, of the 39 pooled samples, 23 samples were positive. Most samples had a low BCoV load (Ct value > 30 Fig. 3), and the results were similar in samples from the two feedlots. BRAV was the second most abundant virus identified after the BoNV and was detected by PCR in 21/39 samples (Fig. 3). Interestingly, we found a significant association of BRAV infection with unaffected control animals, particularly on feedlot 2 (Fig. 3).

BRBV has been infrequently studied as part of the BRD complex. In our study, it was found at low occurrence (4/25 cases and 4/14 unaffected controls) (Fig. 3). Similarly, we detected bovine narmovirus-1 (Bulang virus) in 28/39 samples, without an apparent relationship between the presence of this virus and the occurrence of BRD (Fig. 3).

When the qPCR was used, BoHV-1 was detected in 37/39 of the samples analysed (it was not detected in one pool from BRD affected animals and one pooled sample from non-affected animals). Nine samples had a BoHV-1 Ct value < 25. In the logistic regression, there was a significant association between the Ct values and occurrence of disease on feedlot 1 (Fig. 2; Fig. 3).

### Phylogenetic analysis

#### Bovine nidovirus (BoNV)

**Fig. 4:**
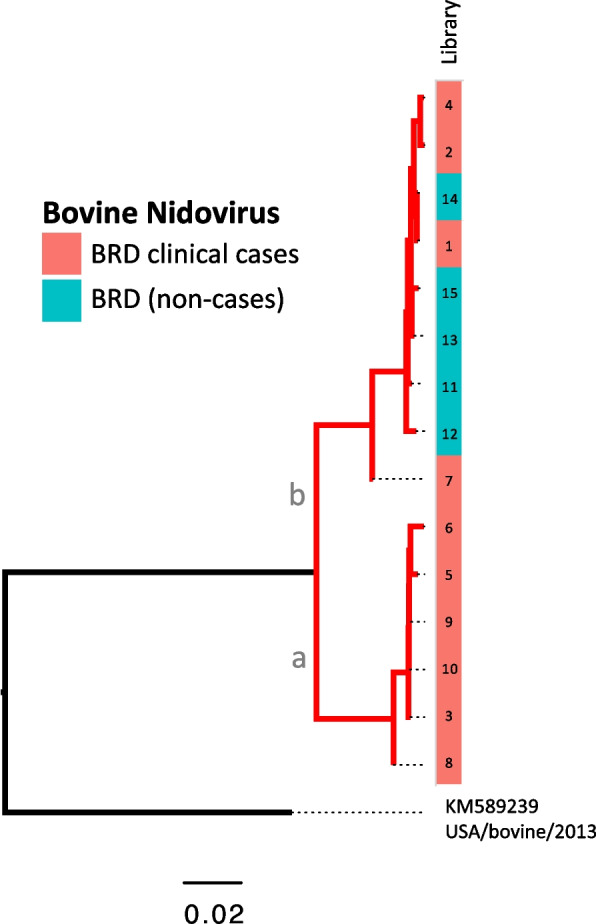
Maximum likelihood phylogeny of BoNV. The tree is midpoint rooted constructed with the complete genome nucleotide sequences. Branch length indicate nucleotide substitutions per nucleotide site. Two distinct branches with sequence from BRD cases and unaffected controls are depicted

BoNV is a single-stranded (ss) positive-sense RNA virus (order *Nidovirales*, family *Tobaniviridae*, *genus Bostovirus*). A complete or near complete genome sequence was obtained from all libraries of cases and unaffected controls. The BoNV from our study shows ~ 91% nucleotide identity with the only BoNV reference genome available in GenBank. The phylogeny of the BoNV in our study shows two distinct clades. Notably, all the samples from unaffected controls belong to clade (b), whereas case samples contain viruses from both (a) and (b) clades (Fig. 4). Bootstrap values of the tree are depicted in Additional file 4.

#### Influenza D virus (IDV)

**Fig. 5:**
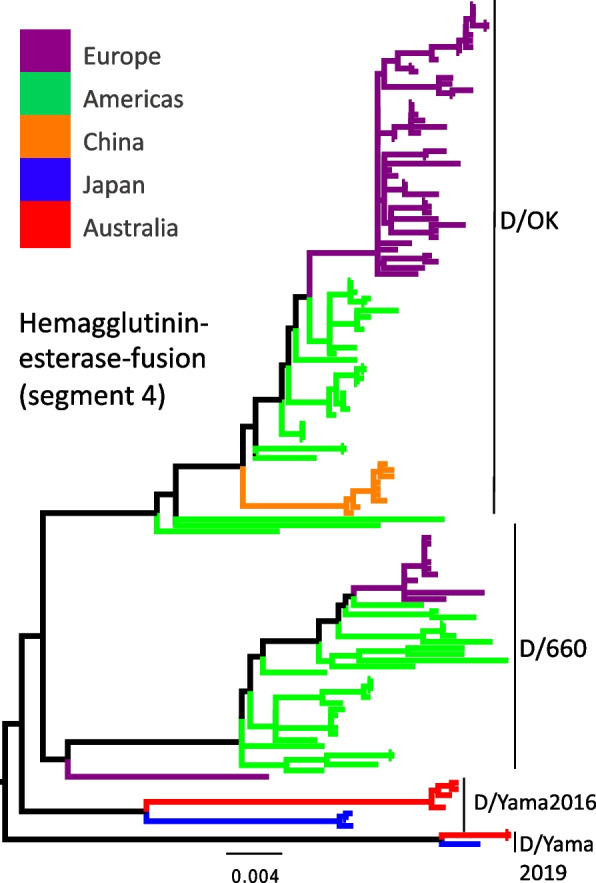
Maximum likelihood phylogeny of influenza D virus hemagglutinin-esterase-fusion protein (segment 4). The colours of the tips in the trees indicate the geographic area. Tree is midpoint rooted. Branch length indicate nucleotide substitutions per nucleotide site. The four different IDV lineages are indicated in the HEF phylogeny. Australian viruses from this study belong to the Japanese lineages D/Yama2016 and D/Yama2019

In contrast to influenza A and B viruses, the genome of IDV comprises seven segments and does not have a neuraminidase protein; rather, it has one surface hemagglutinin-esterase-fusion protein (HEF) [35]. Based on the HEF protein phylogeny, the IDV sequenced from Australian cattle were grouped within the D/Yama 2016 and D/Yama2019 lineages, although the former is relatively divergent from the group (Fig. 5). These lineages have been identified only in Japan, and are divergent from viruses collected in Europe, the Americas and China. Phylogenetic analyses of other segments show a similar grouping (Additional file 5.1). Reference sequences used in the phylogenetic trees of HEF and other segments are depicted in the phylogenies of Additional file 5.2–5.8.

#### Picornaviruses

Three different known species of bovine picornaviruses were identified: BRAV, BRBV virus and EV-E. The phylogenies of these viruses were estimated using a conserved (3D polymerase) and variable (major capsid proteins VP3, VP2 and VP1) coding regions. Bootstrap values of the phylogenies are depicted in Additional file 6.1–6.6.

##### Bovine rhinitis A (BRAV)

**Fig. 6:**
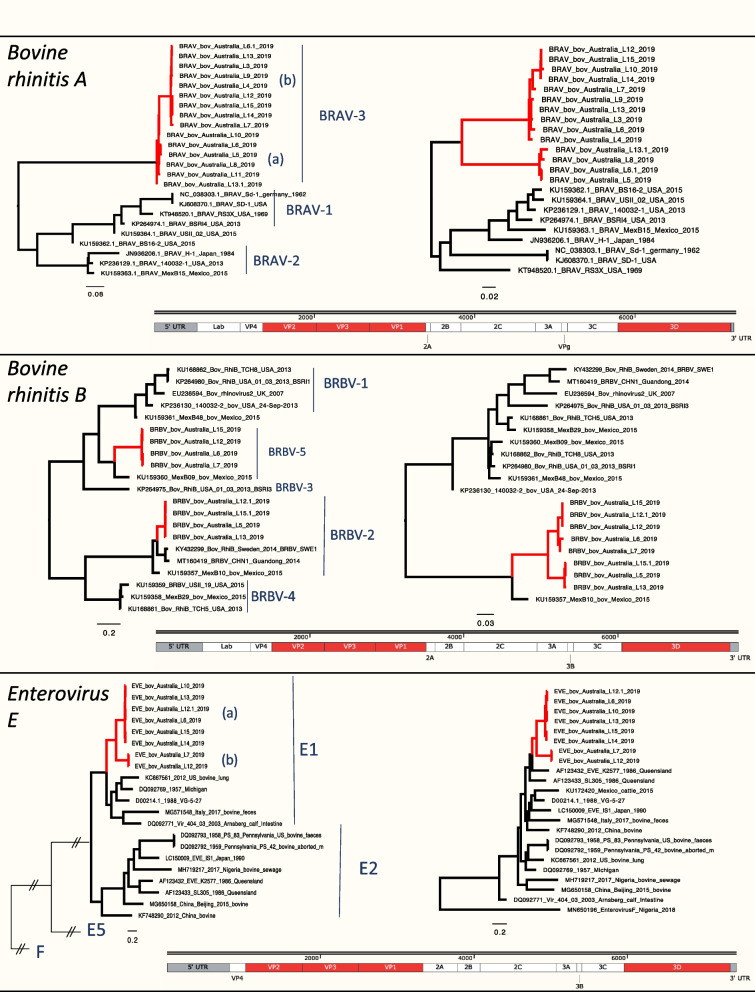
Maximum likelihood, midpoint rooted phylogenetic trees inferred using the nucleotide sequences of bovine rhinitis A virus, bovine rhinitis B virus and enterovirus E. The major capsid phylogeny VP2/VP3/VP1 are on the left of the figure, while the more conserved 3D coding region are on the right of the figure. Viral species are indicated in the capsid phylogenies. The genome structure is depicted at the bottom of the figure, and the genomic regions used to estimate the phylogenies are coloured in red

Two different genotypes have been described and taxonomically named BRAV-1 and BRAV-2. The BRAV sequences from this study were genetically different from the published reference sequence and tentatively form a different BRAV genotype-3 Fig. 6). Overall, 15 complete BRAV viral genomes were obtained from this study, falling in two distinct sub-clusters in the capsid coding region and 3D region. The phylogenetic grouping pattern in 3D and capsid coding region was not consistent across the sequences.

##### Bovine rhinitis B (BRBV)

Eight complete genome sequences were assembled from the pooled metagenomes. Based on the capsid proteins, four viruses belong to the BRBV-5 and four belong to the BRBV-2. In contrast, the phylogeny of 3D grouped all viruses together with a Mexican reference of BRBV-2 group (Fig. 6).

##### Enterovirus E (EV-E)

Viruses sequenced in this study fell into a single group within species EV-E1. Australia viruses collected in 1986 (EV-E2 species) are closely related in the 3D protein sequence phylogeny but not in the capsid coding sequence (Fig. 6).

#### Bovine narmovirus-1 (Bulang virus)

**Fig. 7:**
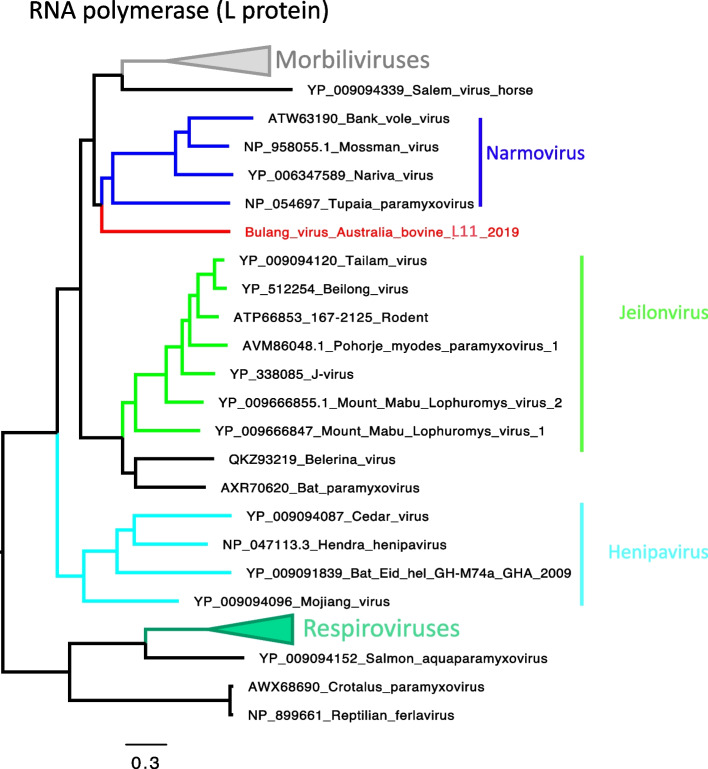
Maximum likelihood phylogeny bovine narmovirus-1 (Bulang virus) L protein. The phylogeny was performed with amino acid sequences. Tree is midpoint rooted. Bulang virus groups within the Narmovirus genus, from small mammals, bats and rodents’ hosts

We obtained the complete genome sequence of a novel paramyxovirus species, provisionally called bovine narmovirus-1 (Bulang virus). Phylogenetically, this paramyxovirus falls within the genus *Narmovirus* that includes Tupaia, Bank vole, Nariva and Mossman viruses, which are viruses sequenced from bats, rodents and small mammals (Fig. 7) [37]*.* The phylogenetic tree of the different protein coding genetic regions of the virus are available in Additional file 7.1–7.6.

### Bacterial taxonomy and abundance

**Fig. 8:**
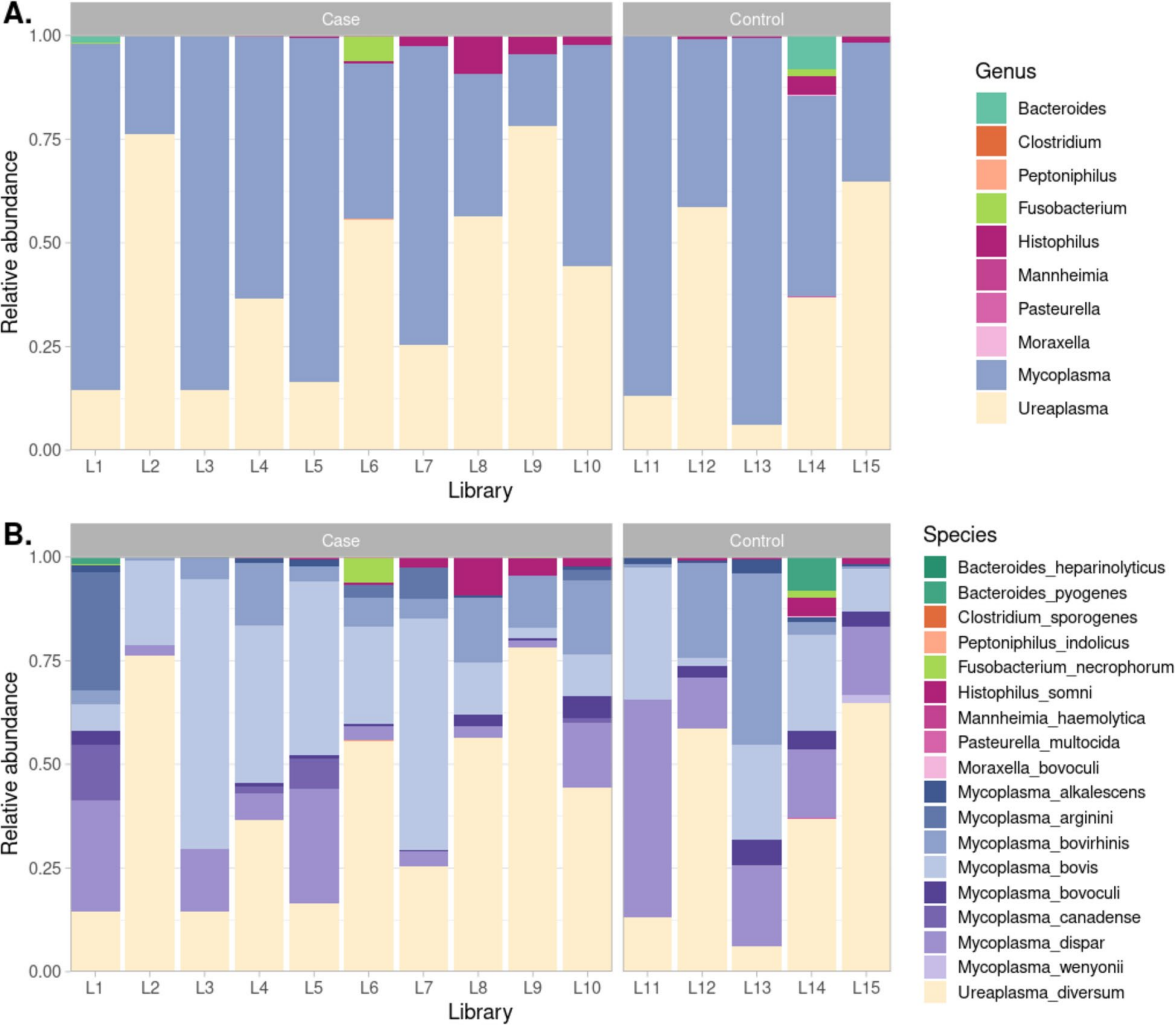
Relative abundance of bacterial species in case and control libraries. **A** Overall genera diversity with unassigned reads removed. Ureaplasma and Mycoplasma are the genera with the highest abundance in all libraries. **B** Relative abundance at the species level. Unassigned sequences were removed. Several mycoplasma species were present in case and control libraries

The relative abundance of bacteria was inferred across taxonomic ranks for all libraries. The *Porphyromonadaceae* family (*n* = 3) and *Moraxella bovoculi* (*n* = 1) were filtered out due to low relative abundance. A total of 18 species from 10 genera and 7 families were retained. *Mycoplasmataceae* form the majority across all libraries, with library L10 having the lowest relative abundance (85%; Fig. 8). *Mycoplasma bovis*, *Mycoplasma bovirhinis*, *Mycoplasma dispar* and *Ureaplasma diversum* were present in all libraries. *Mycoplasma canadense* was present in 4/10 case libraries and none from unaffected controls, *Mycoplasma bovoculi* in the same proportion from both groups of animals (8/10 BRD cases and 4/5 unaffected controls) and *Mycoplasma arginini* in 5 case libraries and no libraries from unaffected ‘control’ libraries. Of the non-Mycoplasmataceae, *Fusobacterium necrophorum* 4/10 cases and 1/5 unaffected controls, *Histophilus somni* 8/10 cases and 4/5 unaffected controls, *Pasteurella multocida* 3/10 cases, 1/5 unaffected controls, *Mannheimia haemolytica* only found in 1/10 cases.

Notably, there were no significant differences between the alpha diversity of the libraries from the 2 groups of animals (Kruskal–Wallis: observed (*p* = 0.289); Shannon (*p* = 0.540); Simpson (*p* = 0.713); Fisher (*p* = 0.270)) (Additional file 8a). Similarly, there were no significant differences between the beta diversity of libraries from BRD affected and unaffected animals (PERMANOVA: *p* = 0.409; Additional file 8b).

### Relative abundance of AMR genes identified in the transcriptome

Six AMR genes were identified in the transcriptome, conferring resistance against tetracycline: *tet*Q, and *tet*H, macrolides: *mef*A and *mph*E, and streptogramin: *vat*E and *msr*E. The β-Lactamase resistant gene bla_TEM-4_ gene was found in all pools; however, the contig containing this gene had a high match with a cloning vector (Cloning vector pBR322), so it was considered a contaminant. The contig in which *erm*(B) gene found in seven libraries was also identified as part of a cloning vector (Cloning vector pZJ23).

**Fig. 9:**
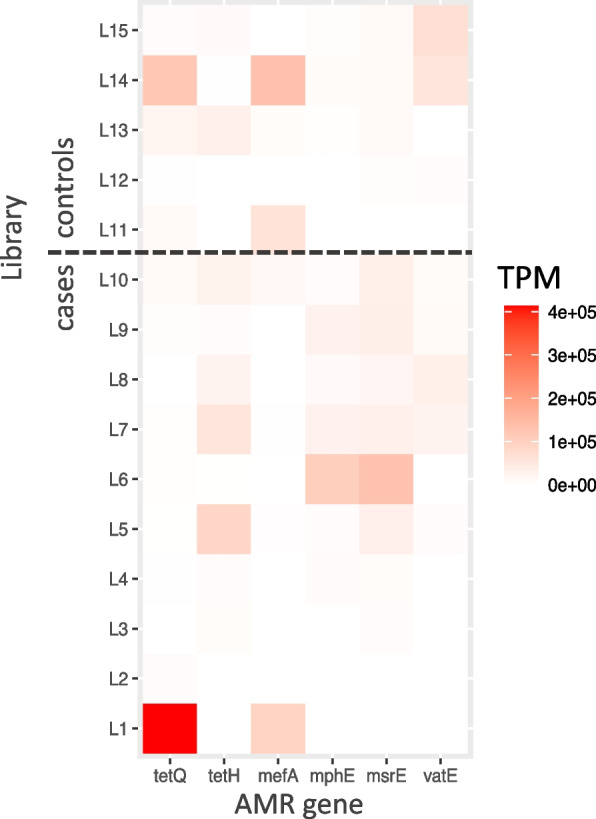
Heatmap depicting the transcripts per million (TPM) relative estimation of AMR gene identified in the transcriptome of animals with BRD (L1-L10) and without BRD (L11-L15)

One case library (L1) had a higher abundance of *tet*Q tetracycline resistance gene, which was also present in unaffected “control” samples. A variable abundance of *tet*H, *mef*A and *vat*E was identified in all libraries, while the abundances of *mph*E and *msr*E were consistently low in samples from unaffected control animals (Fig. 9).

### Eukaryotic pathogens

We identified RNA that was taxonomically classified within the families *Aspergillaceae*, *Ascobolaceae*, *Nectriaceae*, *Trichosporonaceae*, *Wallemiaceae*, *Rhabditidae*, *Strongyloididae*, *Ancylostomatidae*, *Chabertiidae*, *Cooperiidae*, *Babesiidae*, *Theileriidae*, *Sarcocystidae*, *Entamoebidae*, *Hypotrichomonadidae*, *Trichomonadidae* and *Simplicimonadidae*. Families present with higher frequency in the libraries were *Strongyloididae* (*n* = 12), *Entamoebidae* (*n* = 12) and *Trichomonadidae* (*n* = 11). Entamoeba was present in 5 libraries from animals without BRD and only 2/10 libraries from affected animals; *p* = 0.003). Similarly, the presence of *Simplicimonas similis* was associated with “control” libraries (*p* = 0.005). None of the other species or genus found was associated with cases.

## Discussion

We have identified a variety of viruses infecting the upper respiratory tract in Australian feedlot cattle. While some of the viruses identified are known to cause respiratory disease in cattle, this study highlights that there is a variety of other viruses that may have an impact on the occurrence of BRD. Of most note was the high abundance of BoNV in all samples, the presence of IDV, the identification of a new *Paramyxovirus* and the wide diversity of *Mollicutes*.

There are no previous reports of BoNV in cattle in Australia. Globally, the previously published genome of BoNV was assembled from sequences obtained from lung samples of cattle during a severe bovine BRD outbreak in a US feedlot in 2013, but the significance of BoNV in disease was not conclusive [38]. Although it has been detected in metagenomics studies of cattle in both the USA and Canada, this virus has not yet been isolated in culture, and its pathogenesis is unknown [7–9]. Our results contrast the findings of a study of Canadian beef cattle, which detected BoNV in association with controls rather than cases [9]. Further studies should aim at isolating this virus to determine its role in disease.

Influenza D virus has been previously detected in North America, Europe and Asia. Most detections have been in cattle and swine populations, although it can also affect sheep, goats, horses and camels [39–41]. It is believed that cattle are the natural reservoir, and from them, the virus can spill over to swine. Pathogenesis studies have suggested that IDV alone does not cause severe respiratory disease but can replicate in the upper and lower respiratory tract in calves 2–6 weeks old [42, 43], whereas metagenomics studies have found an association with BRD [10]. Although we found no apparent association between the Australian IDV genotypes and BRD, our sample size was small, and our age target were animals older than weaning age. The presence of other pathogens or specific environmental and management characteristics may modulate the potential of IDV as a pathogen.

BCoV was present in most animals sampled in this study, and RNA load was higher in nasal swabs compared to nasopharyngeal swabs. Although BCoV is most often known to cause gastroenteric disease, it has been associated with respiratory disease outbreaks in the past in Australia and Italy [44, 45]. While we did not identify an association between the detection of BCoV and respiratory disease, the specific role for BCoV in the BRD complex may vary depending on the virus strain, susceptibility, management practices and concomitant viral and bacterial infection [46, 47].

BoHV-1 is the etiologic agent of infectious bovine rhinotracheitis and a known pathogen within the BRD complex (39). Although we identified BoHV-1 in 7/10 case libraries and 2/5 control libraries, the abundance estimation is not directly comparable to the abundance estimated for RNA viruses as no genomic DNA was sequenced. However, this virus was detected by qPCR in almost all samples and the viral DNA concentration was high for several animals (Ct < 25). It is not possible to interpret the relevance of these data in the context the BRD complex because a live BoHV-1 vaccine virus had been administered intranasally to these cattle at induction to the feedlot [48].

Picornaviruses such as BRAV, BRBV and EV-E have been infrequently associated with disease outbreaks in livestock. Recent metagenomics studies have revealed that BRAV and BRBV are often found in intensively managed cattle [49, 50]. However, the prevalence of these viruses is largely unknown in Australia. Although our sample size is limited, the abundance of BRAV and its association with healthy animals may challenge a possible contribution of BRAV to the BRD complex.

Two respiratory paramyxoviruses are known to circulate in Australian cattle, namely BRSV and bovine parainfluenza 3 (BPI3; *Bovine respirovirus 3*). While a previous study found that seroprevalence at feedlot induction was for 89% BRSV and 91% for BPI3, we detected BRSV in a low number of samples and PI3 was not detected in this study. The lack of detection may be due to animals exposed at an earlier age, or that seasonal waves of infection affect feedlots, but it is unlikely that this virus is completely absent from the feedlots sampled. BRSV is a known pathogenic virus and plays a role in the BRD complex in many countries. BRSV seroprevalence is high in Australian feedlot cattle [51]. Despite its ubiquitous distribution, genomic sequences data are very scarce with only four complete genome sequences were available in public reference datasets (NCBI GenBank). Therefore, the global genetic diversity of BRSV is largely unknown. We were also able to sequence the complete genome of a novel bovine paramyxovirus, bovine narmovirus-1 (Bulang virus), evolutionary different from other bovine paramyxovirus that was present in 28/39 pooled samples from this study. Although there was no significant association between the presence of the virus and the occurrence of BRD, at this stage, the significance of this virus remains unknown.

Bacterial species identified in this study can be found in the typical microbiome of cattle, although several may contribute to clinical respiratory disease [10]. We found a striking abundance of *Mycoplasma sp.* in most sequenced RNA libraries. All these Mycoplasma species have been detected from healthy and clinically diseased animals in targeted studies or those sequencing the bovine respiratory microbiome [52, 53]. Globally, there has been increasing concern over the rising prevalence of *M. bovis* in dairy and beef cattle operations [54]. In New Zealand, ongoing aggressive efforts to eradicate it after its introduction in 2017 have taken place [55]. In Australian feedlots, the seroprevalence of *M. bovis* at induction was an estimated 3.5%, but 25.3% 6 weeks after induction [56]. Establishing the relationships between detection with different molecular methods, serology and sequencing would also contribute to a better understanding of the impact of *Mycoplasma sp.* in cattle disease.

The transcriptome of BRD cases and non-cases revealed a variable AMR expression against tetracyclines and macrolides. A similar finding was reported in a recent study, which recovered *P. multocida* and *M. haemolytica* from fatal cases of BRD in Australian feedlot cattle. While most *M. haemolytica* isolates were susceptible to all antimicrobials licensed for use in cattle, several *P. multocida* were resistant to tetracycline, tilmicosin, tulathromycin/gamithromycin and ampicillin/penicillin, and some were resistant to tetracycline and macrolides. The AMR genes identified in *P*. *multocida* were aminopenicillins, macrolides *msr*E and *mph*E, and tetracycline resistance genes *tet*H-*tet*R or *tet*Y, which is similar to the tetracycline-macrolide resistance profile identified in this study [11].

The increasing application of metagenomics approaches to study the microbiome and virome has been pivotal in understanding the bacterial diversity associated with diseases with polymicrobial aetiology. Although viruses have an essential role in the respiratory disease complex of all animal species, there has been a paucity of data on the viral component in metagenomics studies. This is due, in part, to the genetic diversity and lack of a common gene marker present in all viruses, which rules out the cheaper and faster approaches to using a limited set of primers to sequence communities (e.g. PCR approaches targeting 16S rRNA gene in bacteria). The current lack of comprehensive reference sequences for all viral species and genotypes in non-human hosts impedes the automation of taxonomic classification of viromes and requires specialised and labour-intensive assembly of divergent viral genomes. The automation of viral metagenomics data analyses tools depends on the creation of a thorough database with livestock viruses collected from different geographic regions to complete a species and genotype database.

There are several challenges and limitations when interpreting microbial genetic data and its association with disease in cross-sectional studies. The microbial diversity observed may vary across different seasons or feedlot operations; therefore, it is only representative of the animals present at the feedlot at the time of sampling. Causation must be carefully assessed by the isolation of the pathogen candidate and followed up with reproducible clinical outcome with experimental studies. Even in cases where this can be achieved, different experimental studies may yield different results based on the specific genotype or variant used as the inoculum. Cross-sectional studies may therefore guide a deeper understanding of disease association, which may be followed up with observational longitudinal as well as experimental studies. The final outcome depends on complex interactions that are influenced by the timing of infection, the virulence of different virus strains, interactions with other viruses and microorganisms and a wide range of animal hosts, livestock management and environmental factors. For example, the failure to detect viruses such as BVDV and BoHV-1 at a single time point from a modest number of samples should not be misinterpreted and reduce their proven capacity as respiratory pathogens. Instead, the detection of novel agents should be used as a signal to investigate whether these additional viruses could also play a role in the BRD complex.

With respect to the consensus genomes obtained in this study, the quality of the genomes is subject to assembly errors inherent in metagenome-assembled genomes. This becomes particularly relevant in libraries where we identified different genotypes and variants (for example BoNV, IDV, BRAV and BRBV consensus sequence). For example, if more than one closely related virus was present in a sample, the assembly process could fail or could produce a chimeric consensus sequence [57]. Despite this, the consensus sequences from related genotype assemblies in our study were consistent across different libraries supporting a reasonable accuracy of the metagenome-assembled genomes. Diversity analysis in pooled libraries should also be interpreted with caution, provided that samples were classified based on the BRD or non-BRD status, but not by other variables (i.e., age, sampling time).

## Conclusion

We have found a variety of viral and bacterial species in the respiratory tract of feedlot cattle from two feedlots in Australia. From an Australian perspective, we described three previously unrecognised viral species in addition to providing sequence data for known ones. More extensive sample collections, experimental work with recently recognised viruses, automated sequencing and efficient bioinformatic analyses can each contribute to securing information that will provide a better understanding of the role of the infectome in BRD.

## Supplementary Information

**Additional file 1**. Samples collected and pooled for qPCR analysis and sequencing.

**Additional file 2:** **Table.** Primers and probes used to determine viral abundance through qRT-PCR and qPCR.

**Additional file 3**: **Table.** Contigs taxonomically classified as Bovine and Porcine Rotavirus and their amino acid identity to published reference.

**Additional file 4**. Phylogenetic tree of BoNV depicting bootstrap values.

**Additional file 5**. Overview of Influenza D virus’ segments phylogenies. **5.1.** Maximum likelihood phylogenies of the seven segments of Influenza D virus. **5.2.** Influenza D virus- segment 1. **5.3.** Influenza D virus- segment 2. **5.4.** Influenza D virus- segment 3. **5.5.** Influenza D virus- segment 4. **5.6.** Influenza D virus - segment 5. **5.7.** Influenza D virus- segment 6. 5.8- Influenza D virus – segment 7.

**Additional file 6. **Phylogenies with bootstrap values of BRAV, BRBV, EV-E capsid (VP2,VP3 and VP1) and 3D polymerase. **6.1.** Bovine rhinitis A virus – capsid (VP2,VP3 & VP1). **6.2.** Bovine rhinitis A virus– 3D polymerase. **6.3.** Bovine rhinitis B virus – capsid (VP2,VP3 & VP1). **6.4.** Bovine rhinitis B virus– 3D polymerase. **6.5.** Enterovirus E– capsid (VP2,VP3 & VP1). **6.6.** Enterovirus – 3D polymerase.

**Additional file 7. **Overview of Bovine narmovirus-1 (Bulang virus) phylogenies. **Fig 7.1.** Maximum likelihood phylogenies of Bovine narmovirus-1 (Bulang virus). **7.2.** Bovine narmovirus-1 (Bulang virus)– L protein. **7.3.** Bovine narmovirus-1 (Bulang virus)– Nucleoprotein. **7.4.** Bovine narmovirus-1 (Bulang virus)– Matrix. **7.5.** Bovine narmovirus-1 (Bulang virus)– Phosphoprotein. **7.6.** Bovine narmovirus-1 (Bulang virus)– Fusion protein.

**Additional file 8:** **Fig.** Alpha and beta diversity of the metatranscriptomic sequence libraries obtained from animals with clinical bovine respiratory disease (cases) and without clinical disease (controls). Fig(a): The alpha diversity computed using different methods. Fig(b). NMDS plot using Bray-Curtis dissimilarities between samples in cases and controls.

## Data Availability

The sequenced libraries (short reads) were deposited at NCBI BioSample accession numbers 24043620- 24,043,634. The consensus sequences have been submitted to NCBI GenBank accession numbers: ON330449-ON330463, ON861830-ON861833, OP020132-OP020177.
