# Supplementary material for: Expanding the range of the respiratory infectome in Australian feedlot cattle with and without respiratory disease using metatranscriptomics
Source: Microbiome. 2023 Jul 25;11:158. doi: 10.1186/s40168-023-01591-1 (PMC10367309; doi:10.1186/s40168-023-01591-1)
Supplement: Supplementary file 2 — Additional file 1. Samples collected and pooled for qPCR analysis and sequencing. [file 40168_2023_1591_MOESM1_ESM.pdf]

Additional file 2

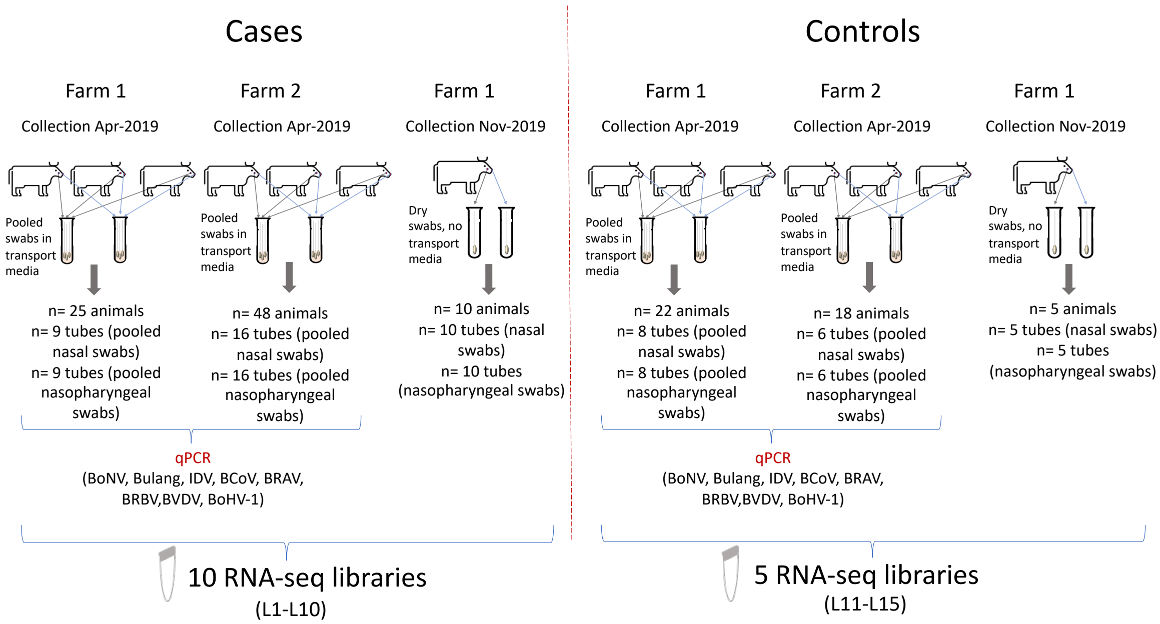

Sample collection in farms 1 and 2 of cases and controls. Samples collected in April of 2019 were pooled (3 nasal swabs in one tube and 3 nasopharyngeal swabs from the same 3 animals in a separate tube). All these pooled samples were subsequently analyzed using qPCR for eight viruses of interest. Pooled samples from cases and from controls from all sampling schemes were used to construct the sequence libraries.
