# Supplementary material for: Expanding the range of the respiratory infectome in Australian feedlot cattle with and without respiratory disease using metatranscriptomics
Source: Microbiome. 2023 Jul 25;11:158. doi: 10.1186/s40168-023-01591-1 (PMC10367309; doi:10.1186/s40168-023-01591-1)
Supplement: Supplementary file 3 — Additional file 2: Table. Primers and probes used to determine viral abundance through qRT-PCR and qPCR. [file 40168_2023_1591_MOESM2_ESM.pdf]

Additional File 1:

Table: Primers and probes used to determine viral abundance through qRT-PCR and qPCR.

Target virus		Primer/Probe sequence 5′–3′
Bovine Nidovirus	F	TCGCCCACGCTTAACGA
	R	GGCATTGAGCAGCGAYTCA
	Pr	CCAAATTTAAGGCGATGCTTGCTTCCG FAM-BHQ1
Bovine narmovirus-1 (Bulang virus)	F	GGATTGTTACCAAGAGGATCATGAT
	R	TATCCAGCATCCCTGCTATGC
	Pr	CCATAATAATACCTCGCGCTGCACATGAGA Qusar 670 BHQ2
Influenza D virus	F	CCCCRATAGGATCAATGTCGAT
	R	TTCTCCCCCTATGATTCCATTYTC
	Pr	CAATTGAAGCCAAAATYCTTGACCGGG TAMARA BHQ2
Bovine Coronavirus	F	GCGTCCAAAGGCTATATTGCTAA
	R	CCCAACATTTGGATTCTGACATAA
	Pr	TGCCTTTCAACAGGTATT FAM-MGB
Bovine Rhinitis A (adapted from Kishimoto et al., 2017) [1]	F	CGGTGTAGCGACGACAACA
	R	TGGGCATCCTTAGTCTCCCA
	Pr	GGCCTTGGGACRCCCTCTCCA Quasar670/BHQ2
Bovine Rhinitis B (adapted from Kishmitot M et al., 2017) [1]	F	GAACGCGATTGTGTYYTAGGG
	R	CGCMACTGAGGTTAGCTTCTCC
	Pr	CTGTCCTTTGCACGGCGTGGC Fam/BHQ1
Bovine Viral Diarrhea From [2]	F	BVD190-F GRA GTC GTC ART GGT TCG AC un_labelled_Primer
	R	V326 TCA ACT CCA TGT GCC ATG TAC un_labelled_Primer
	Pr	TQ-pesti TGC YAY GTG GAC GAG GGC ATG C Fam-BHQ1
Bovine alphaherpesvirus-1. From [3]	F	gB-F TGT GGA CCT AAA CCT CAC GGT un_labelled_Primer
	R	gB-R A GTC GAG CAG ACC CGT GTC un_labelled_Primer
	Pr	BoHV1-P AGG ACC GCG AGT TCT TGC CGC FAM-BHQ1

Cycling parameters Reverse transcription step involving one cycle at 45°C for 10 min. The PCR cycling conditions included an RT inactivation/initial denaturation of 95°C for 10 min followed by amplification with 45 cycles of 95°C for 15 sec and 60°C for 45 sec.

References:

1. Kishimoto M, Tsuchiaka S, Rahpaya SS, Hasebe A, Otsu K, Sugimura S, Kobayashi S, Komatsu N, Nagai M, Omatsu T *et al*: **Development of a one-run real-time PCR detection system for pathogens associated with bovine respiratory disease complex**. *J Vet Med Sci* 2017, **79**(3):517-523.
2. Hoffmann B, Depner K, Schirrmeier H, Beer M: **A universal heterologous internal control system for duplex real-time RT-PCR assays used in a detection system for pestiviruses**. *J Virol Methods* 2006, **136**(1-2):200-209.
3. Wang J, O'Keefe J, Orr D, Loth L, Banks M, Wakeley P, West D, Card R, Ibata G, Van Maanen K *et al*: **Validation of a real-time PCR assay for the detection of bovine herpesvirus 1 in bovine semen**. *J Virol Methods* 2007, **144**(1-2):103-108.
