# Supplementary material for: Expanding the range of the respiratory infectome in Australian feedlot cattle with and without respiratory disease using metatranscriptomics
Source: Microbiome. 2023 Jul 25;11:158. doi: 10.1186/s40168-023-01591-1 (PMC10367309; doi:10.1186/s40168-023-01591-1)
Supplement: Supplementary file 4 — Additional file 3: Table. Contigs taxonomically classified as Bovine and Porcine Rotavirus and their amino acid identity to published reference. [file 40168_2023_1591_MOESM3_ESM.pdf]

Additional file 3.

Table. Contigs taxonomically classified as Bovine and Porcine Rotavirus and their amino acid identity to published reference.

Rotavirus segment	Amino acid identity to closest reference	Reference accession number	Reference length	Contig length
Rotavirus VP7 (porcine B)	70.78%	BAI48757.1	242	465
Rotavirus VP1 (bovine C)	100	BAO73924.1	1090	317
Rotavirus NSP4 (bovine B)	82.22	BAW98444.1	208	271
