# Supplementary material for: Expanding the range of the respiratory infectome in Australian feedlot cattle with and without respiratory disease using metatranscriptomics
Source: Microbiome. 2023 Jul 25;11:158. doi: 10.1186/s40168-023-01591-1 (PMC10367309; doi:10.1186/s40168-023-01591-1)
Supplement: Supplementary file 5 — Additional file 4. Phylogenetic tree of BoNV depicting bootstrap values. [file 40168_2023_1591_MOESM4_ESM.pdf]

Additional file 4: Phylogenetic trees depicting bootstrap values

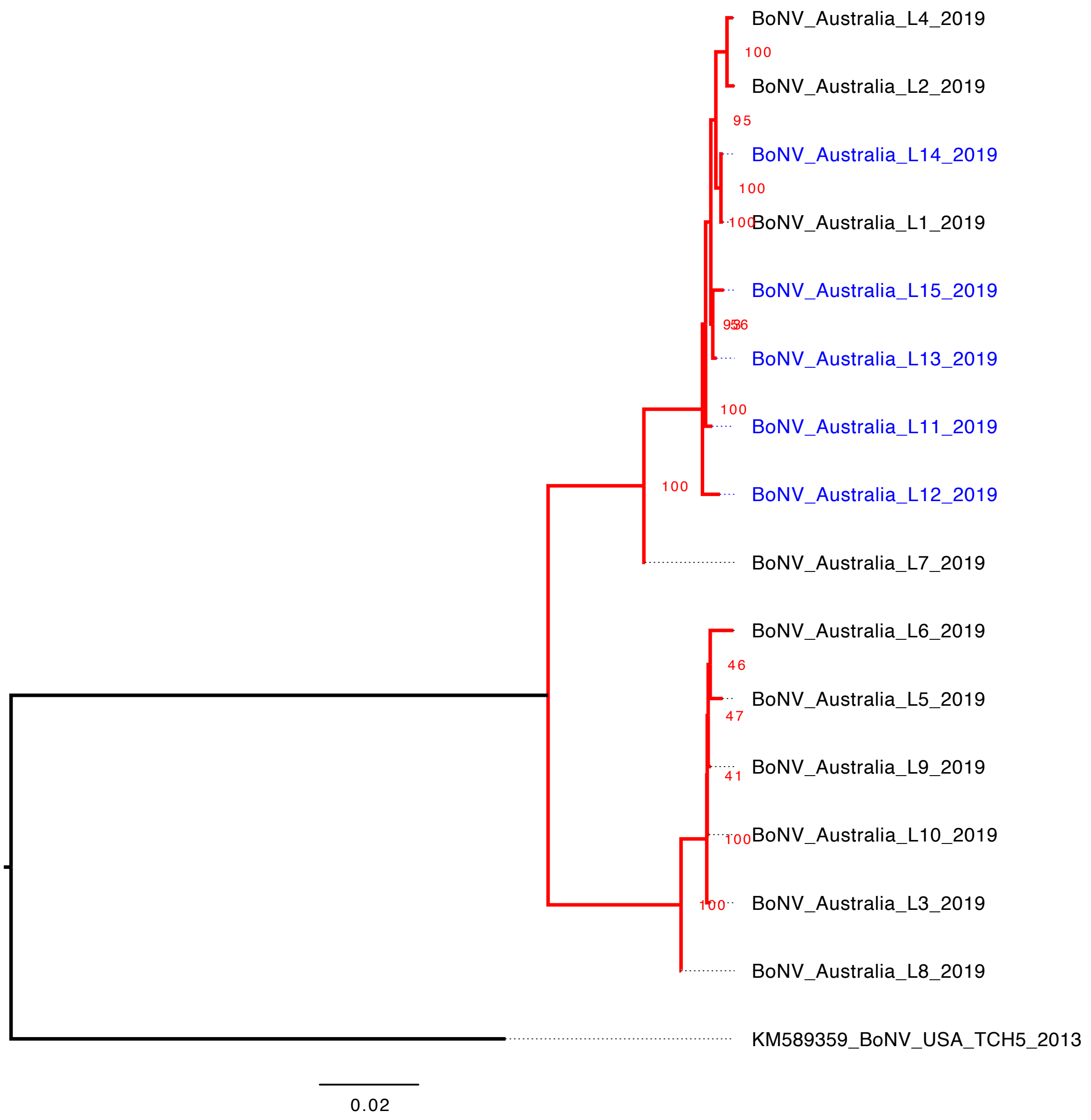

Fig. Maximum likelihood phylogenetic tree of bovine nidovirus (using whole genome).
