# Supplementary material for: Expanding the range of the respiratory infectome in Australian feedlot cattle with and without respiratory disease using metatranscriptomics
Source: Microbiome. 2023 Jul 25;11:158. doi: 10.1186/s40168-023-01591-1 (PMC10367309; doi:10.1186/s40168-023-01591-1)

Additional file 5. Overview of Influenza D virus' segments phylogenies

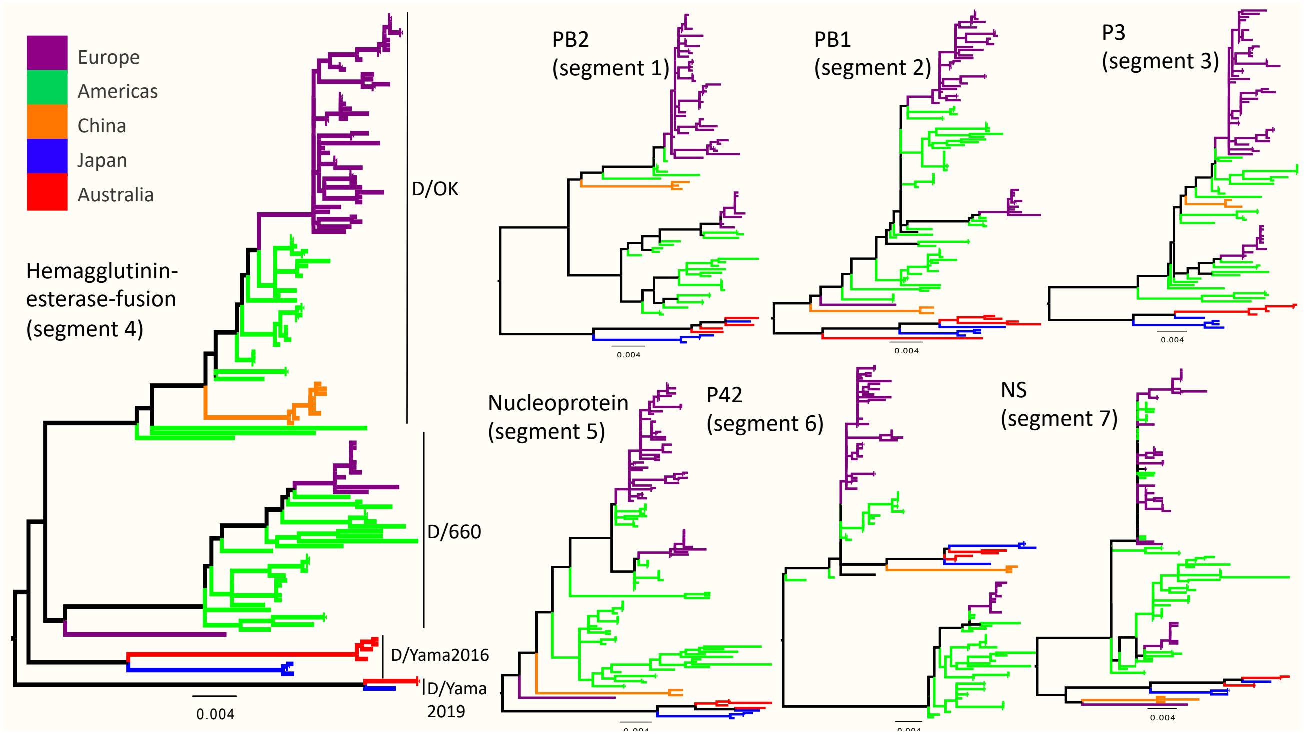

Additional fig 5.1. Maximum likelihood phylogenies of the seven segments of Influenza D virus. The colours of the tips in the trees indicate the geographic area. Trees are midpoint rooted. Branch length indicate nucleotide substitutions per nucleotide site. The four different IDV lineages are indicated in the HEF phylogeny. Australian viruses from this study belong to the Japanese lineages D/Yama2016 and D/Yama2019.

5.2- Influenza D virus- segment 1

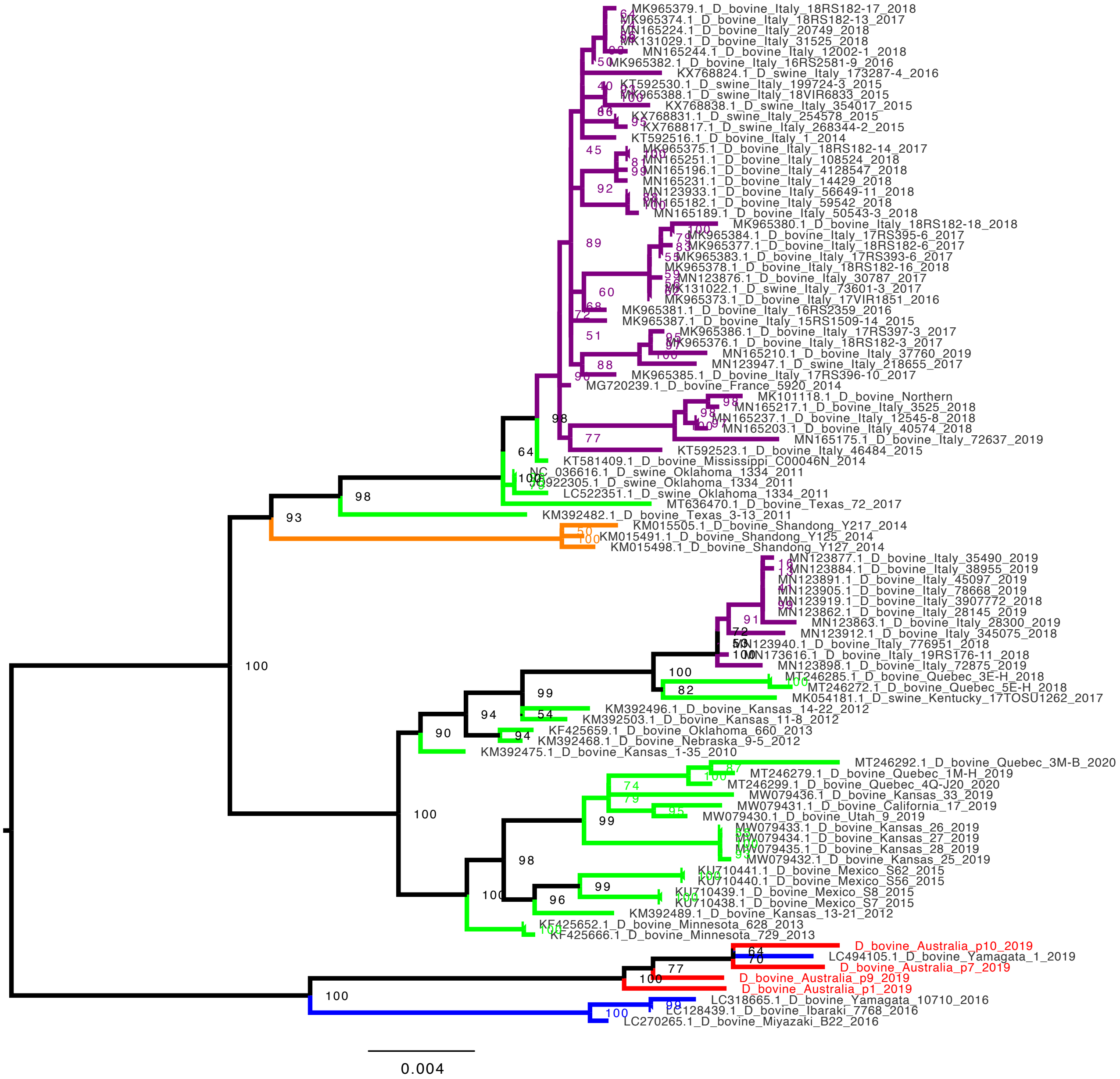

5.3- Influenza D virus- segment 2

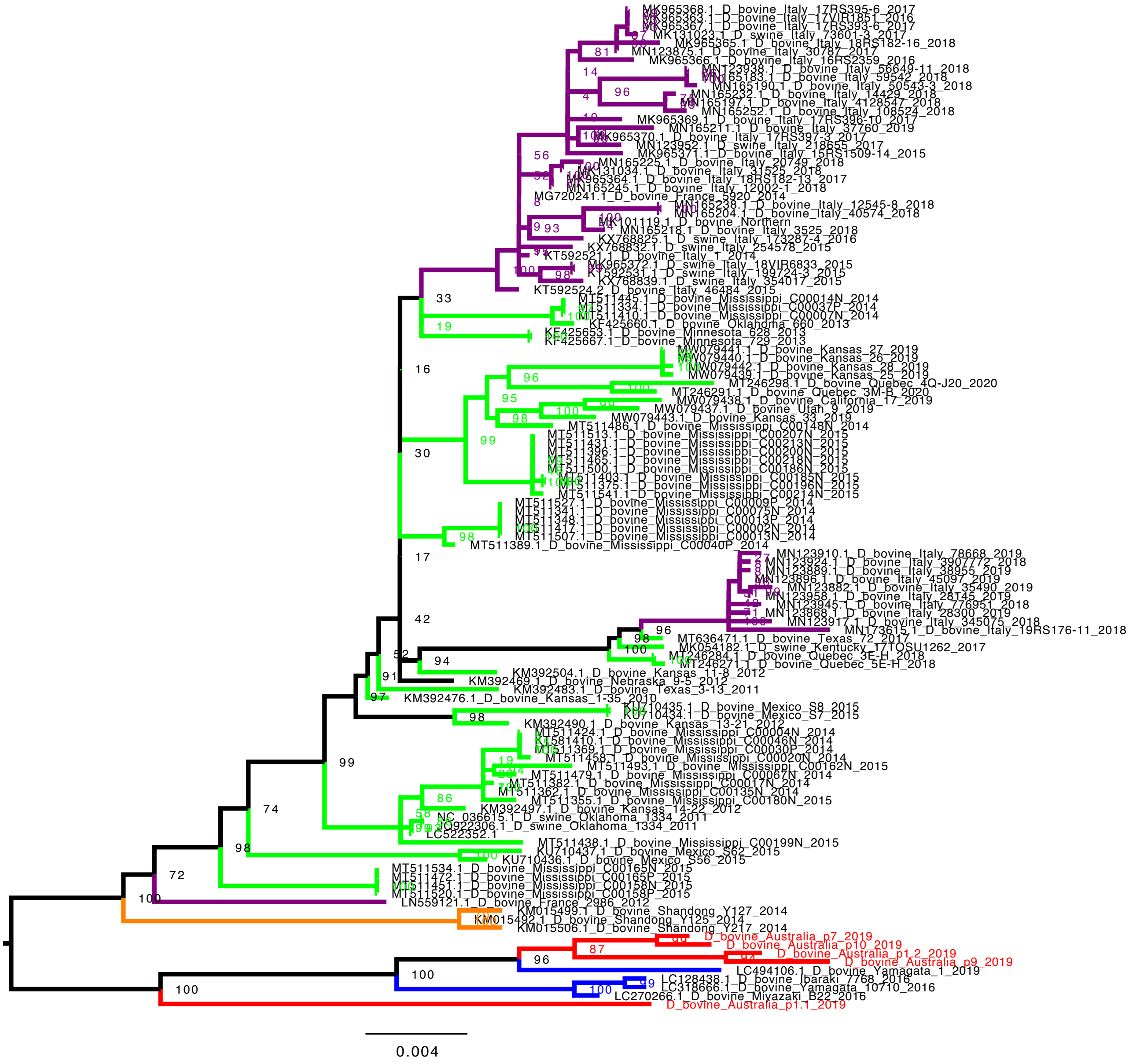

5.4- Influenza D virus- segment 3

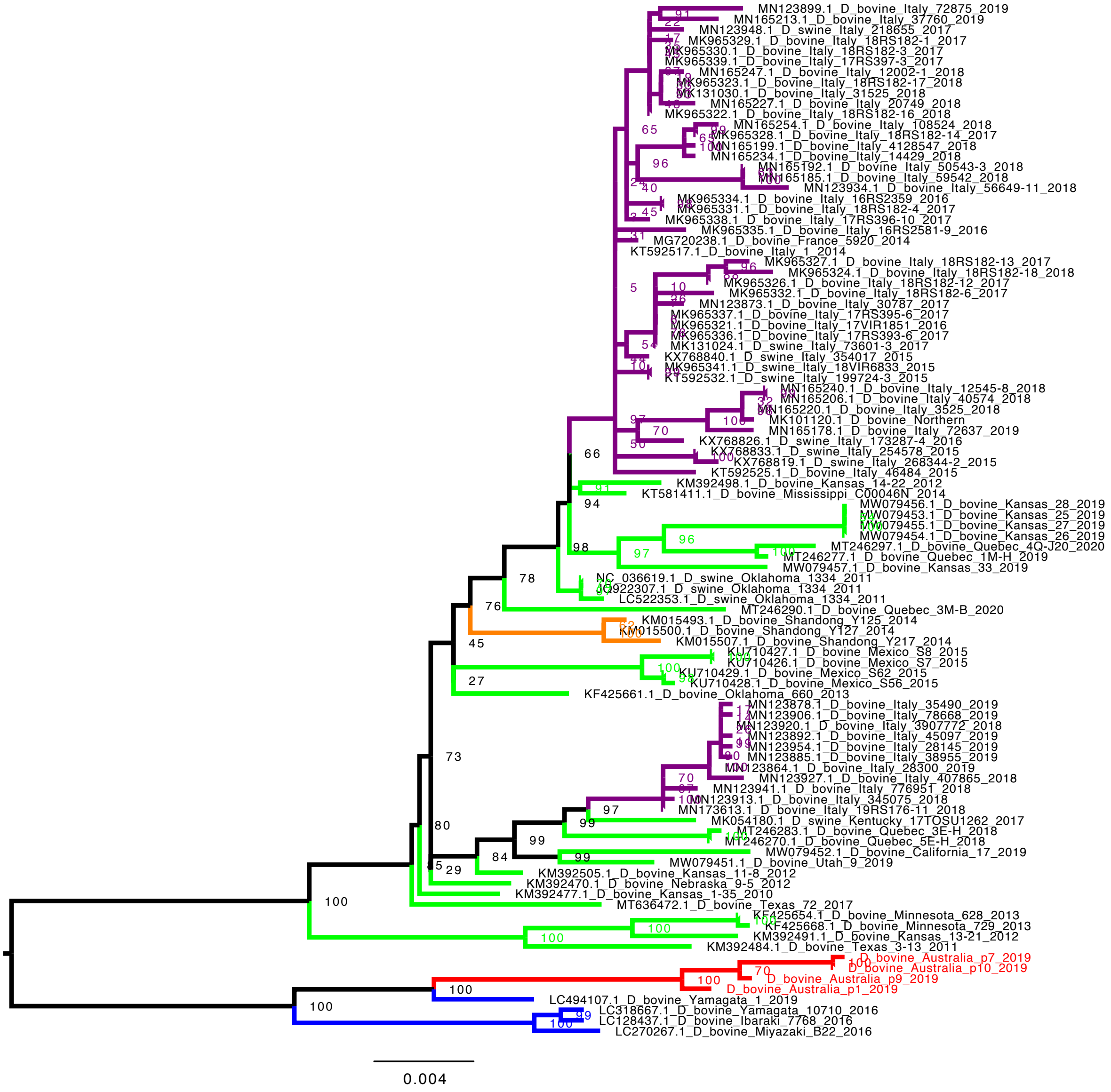

5.5- Influenza D virus- segment 4

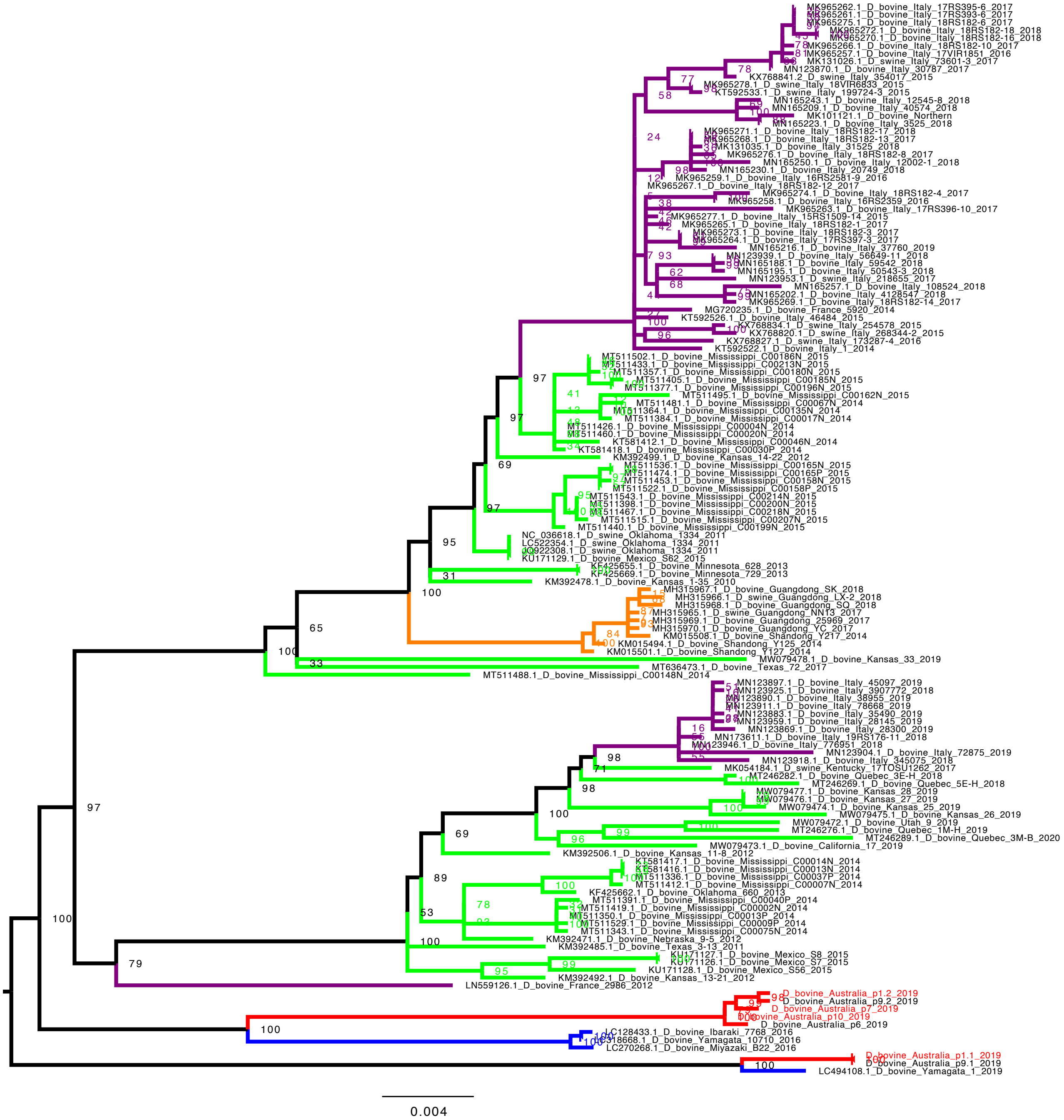

5.6- Influenza D virus - segment 5

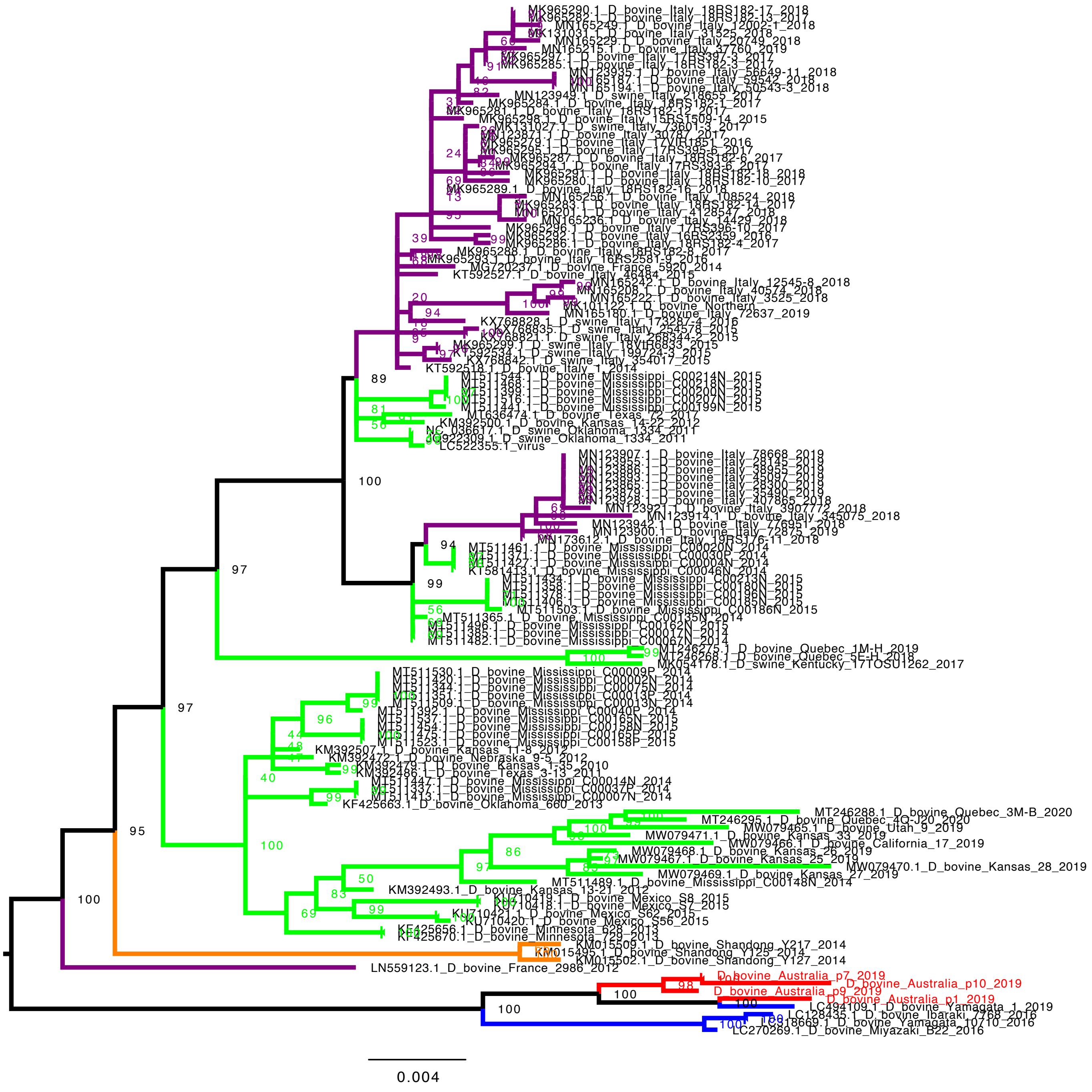

5.7- Influenza D virus- segment 6

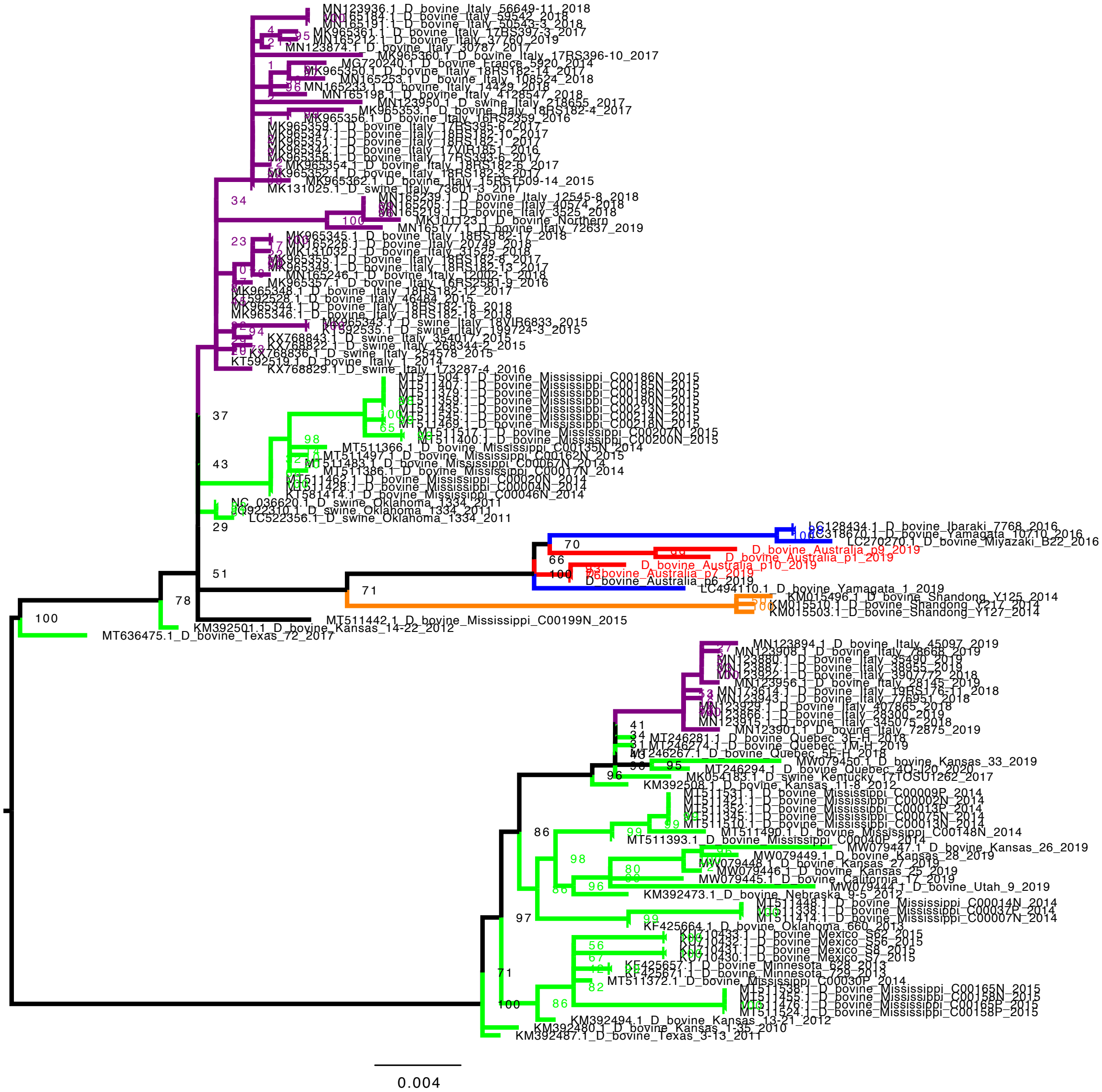

5.8- Influenza D virus – segment 7

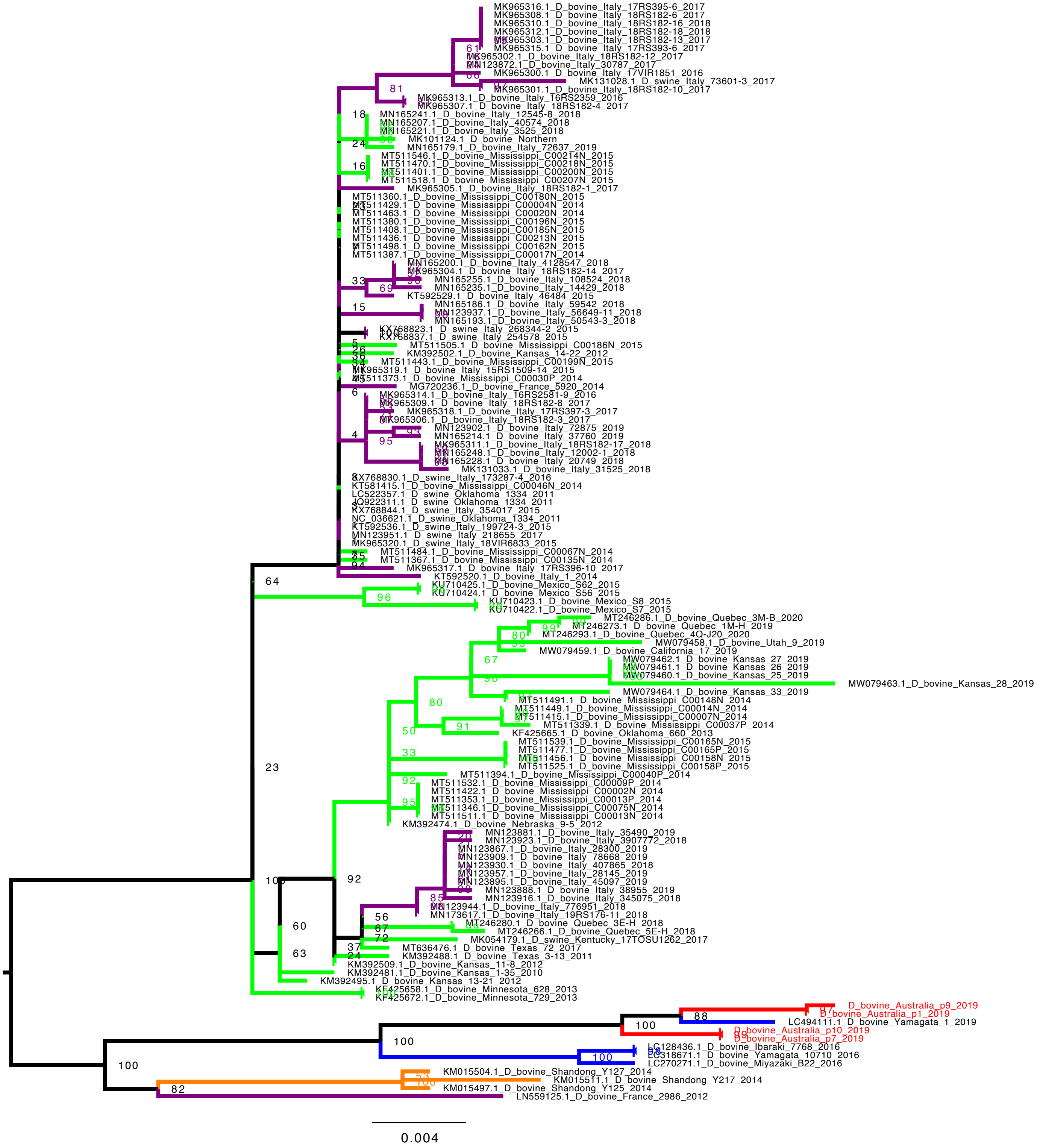

Supplement: Supplementary file 6 — Additional file 5. Overview of Influenza D virus’ segments phylogenies. 5.1. Maximum likelihood phylogenies of the seven segments of Influenza D virus. 5.2. Influenza D virus- segment 1. 5.3. Influenza D virus- segment 2. 5.4. Influenza D virus- segment 3. 5.5. Influenza D virus- segment 4. 5.6. Influenza D virus - segment 5. 5.7. Influenza D virus- segment 6. 5.8- Influenza D virus – segment 7. [file 40168_2023_1591_MOESM5_ESM.pdf]
