# Supplementary material for: Expanding the range of the respiratory infectome in Australian feedlot cattle with and without respiratory disease using metatranscriptomics
Source: Microbiome. 2023 Jul 25;11:158. doi: 10.1186/s40168-023-01591-1 (PMC10367309; doi:10.1186/s40168-023-01591-1)

Additional file 6- **Phylogenies with bootstrap values of BRAV, BRBV, EV-E capsid (VP2, VP3 and VP1) and 3D polymerase.**

6.1 Bovine rhinitis A virus – capsid (VP2,VP3 & VP1)

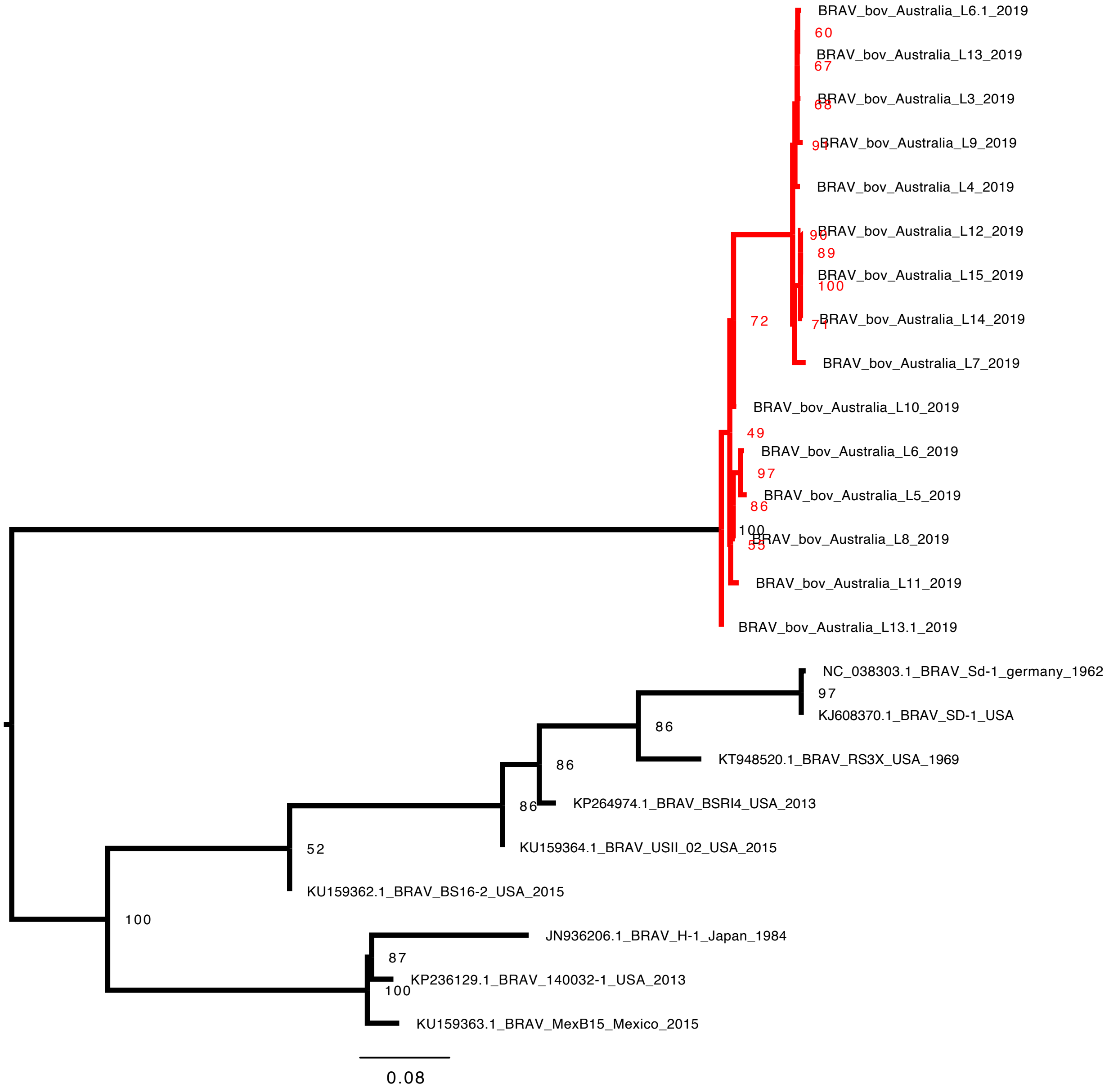

6.2. Bovine rhinitis A virus– 3D polymerase

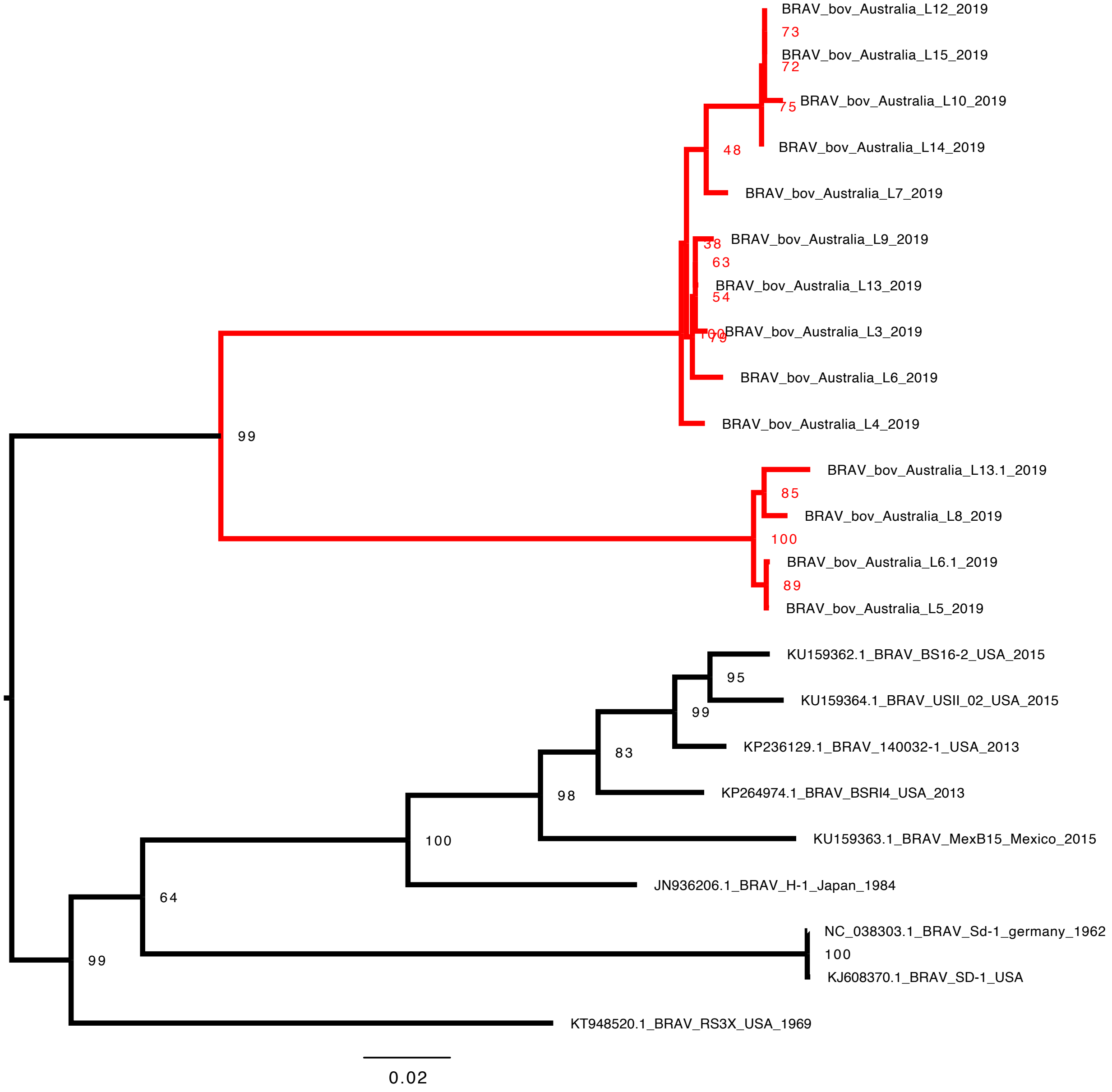

6.3 Bovine rhinitis B virus – capsid (VP2,VP3 & VP1)

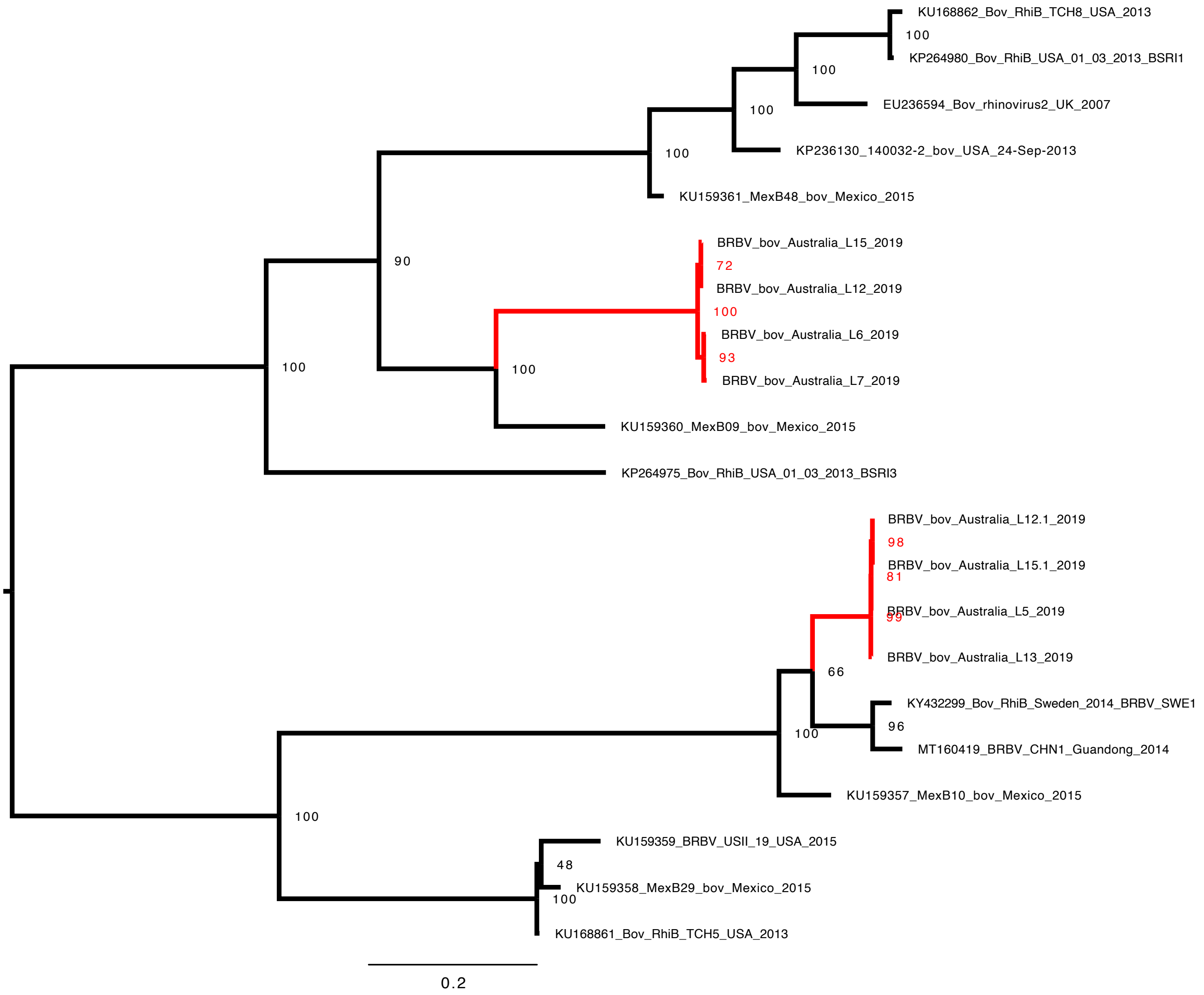

6.4 Bovine rhinitis B virus– 3D polymerase

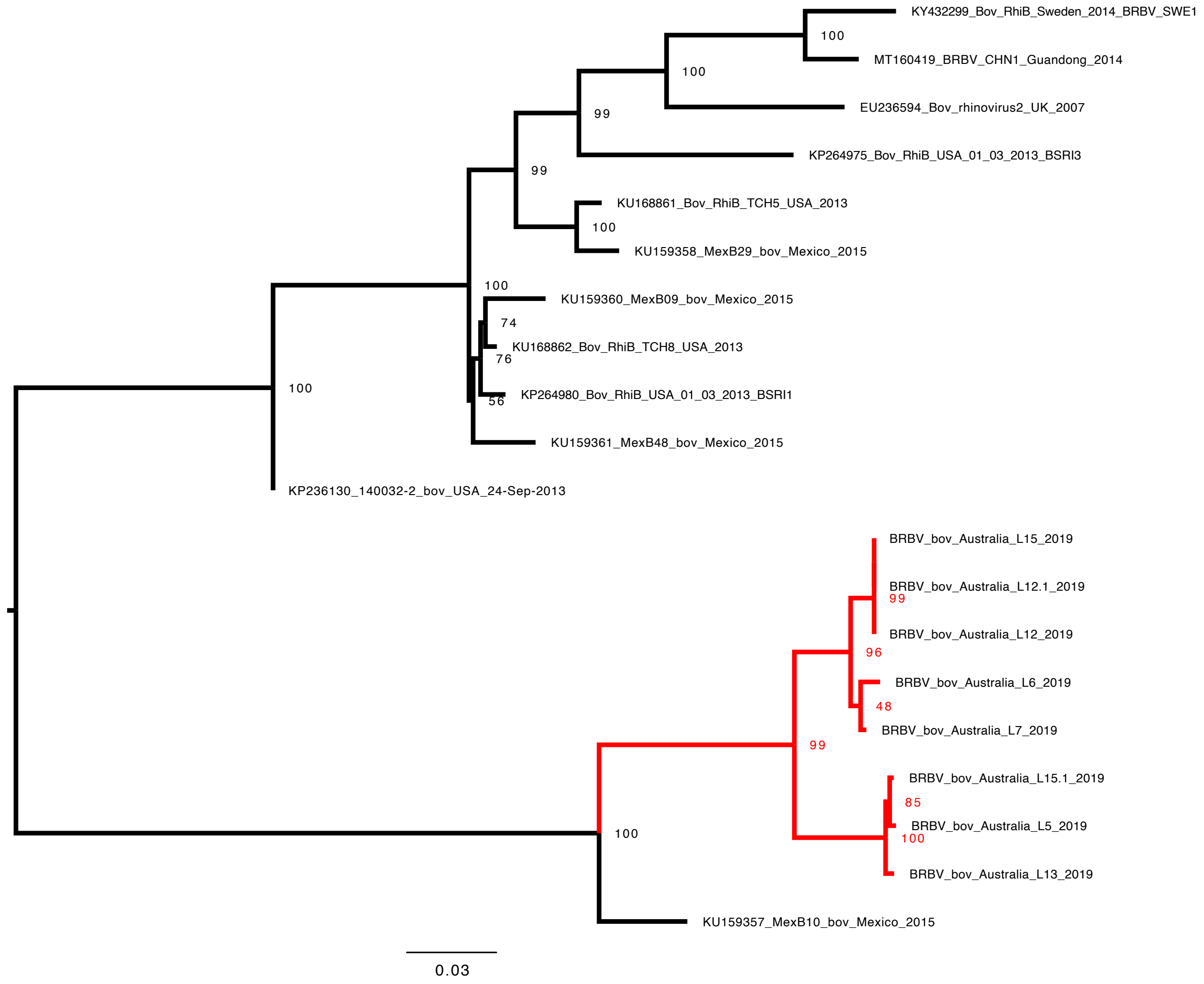

6.5 Enterovirus E– capsid (VP2,VP3 & VP1)

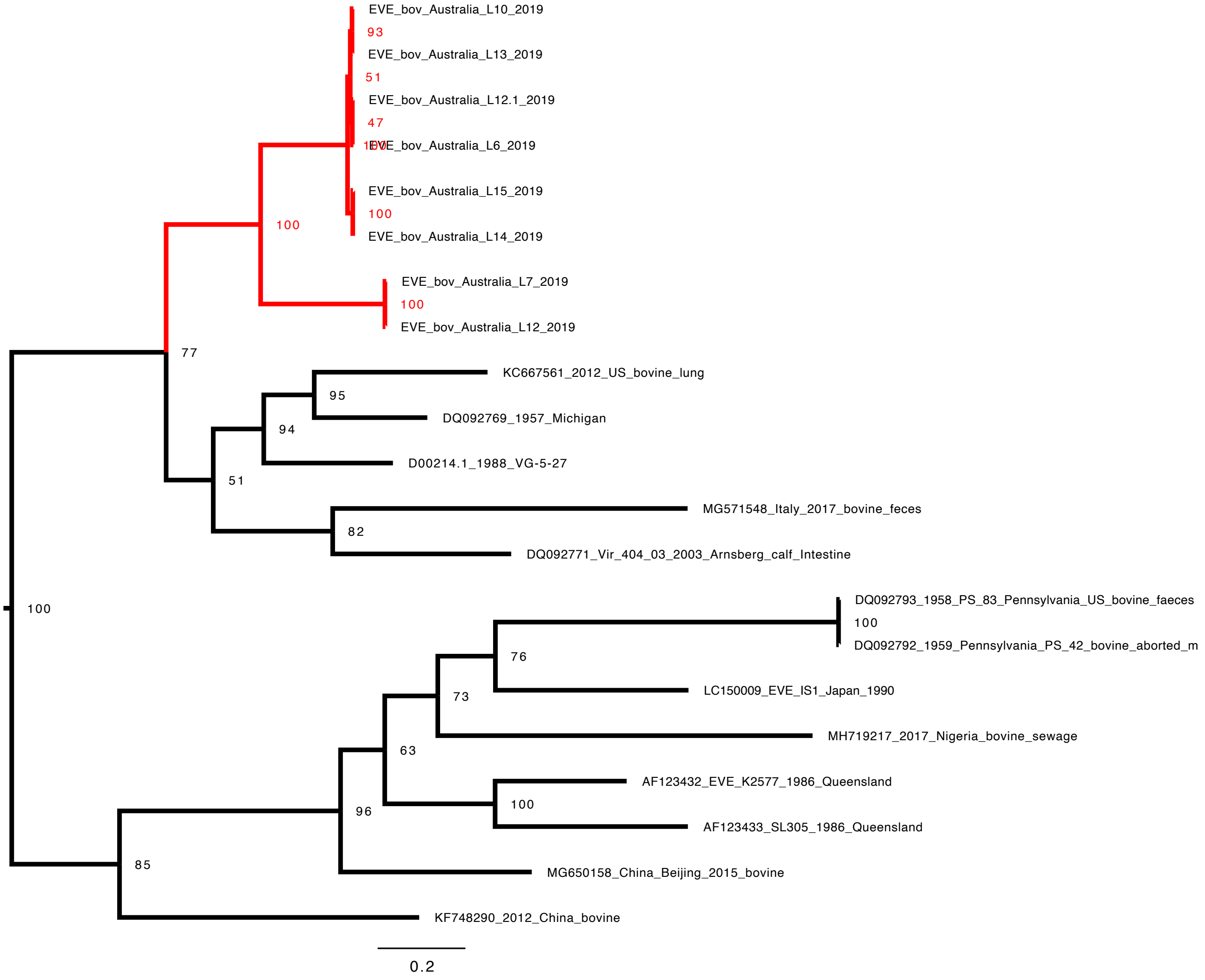

6.6 Enterovirus – 3D polymerase

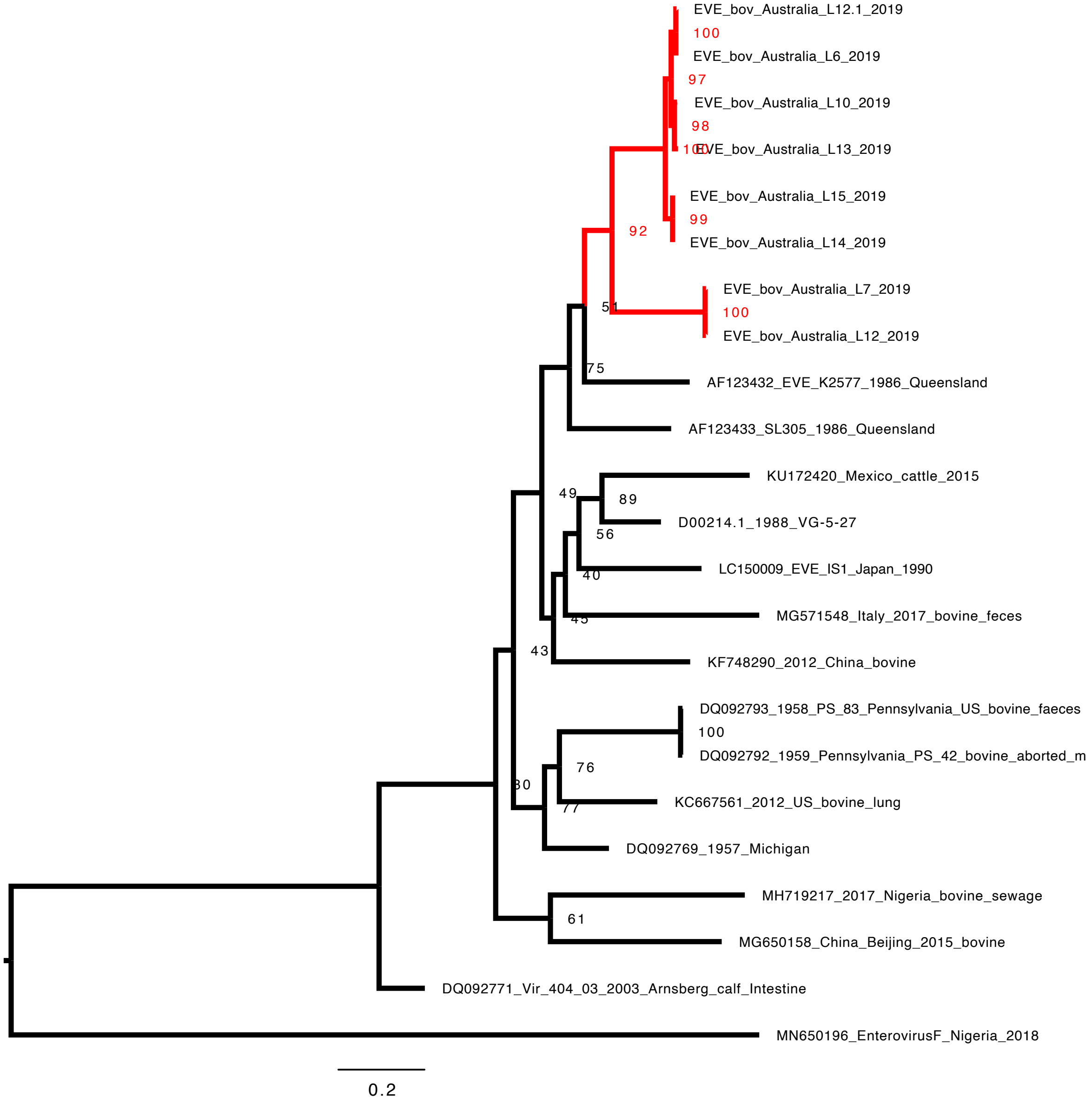

Supplement: Supplementary file 7 — Additional file 6. Phylogenies with bootstrap values of BRAV, BRBV, EV-E capsid (VP2,VP3 and VP1) and 3D polymerase. 6.1. Bovine rhinitis A virus – capsid (VP2,VP3 & VP1). 6.2. Bovine rhinitis A virus– 3D polymerase. 6.3. Bovine rhinitis B virus – capsid (VP2,VP3 & VP1). 6.4. Bovine rhinitis B virus– 3D polymerase. 6.5. Enterovirus E– capsid (VP2,VP3 & VP1). 6.6. Enterovirus – 3D polymerase. [file 40168_2023_1591_MOESM6_ESM.pdf]
