# Supplementary material for: Expanding the range of the respiratory infectome in Australian feedlot cattle with and without respiratory disease using metatranscriptomics
Source: Microbiome. 2023 Jul 25;11:158. doi: 10.1186/s40168-023-01591-1 (PMC10367309; doi:10.1186/s40168-023-01591-1)

Additional file 7. Overview of Bovine narmovirus-1 (Bulang virus) phylogenies

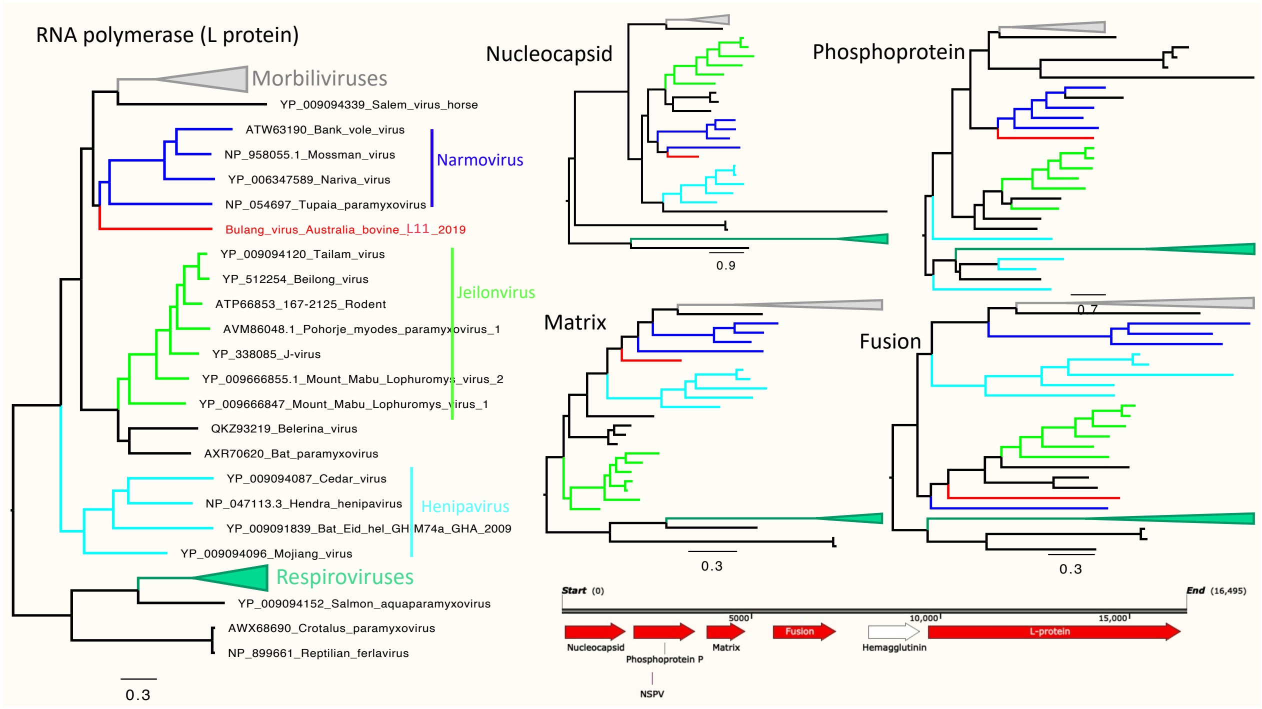

Fig 7.1. *Maximum likelihood phylogenies of Bovine narmovirus-1 (Bulang virus). All phylogenies were performed with the amino acid sequences. Trees are midpoint rooted. Bovine narmovirus 1 groups within the Narmovirus genus, from small mammals, bats and rodents' hosts. Fusion protein phylogeny shows a different grouping. The genomic organization of the virus is shown at the bottom of the figure, the protein coding segments used to construct the phylogenies are coloured in red.*

7.2. Bovine narmovirus-1 (Bulang virus)– L protein

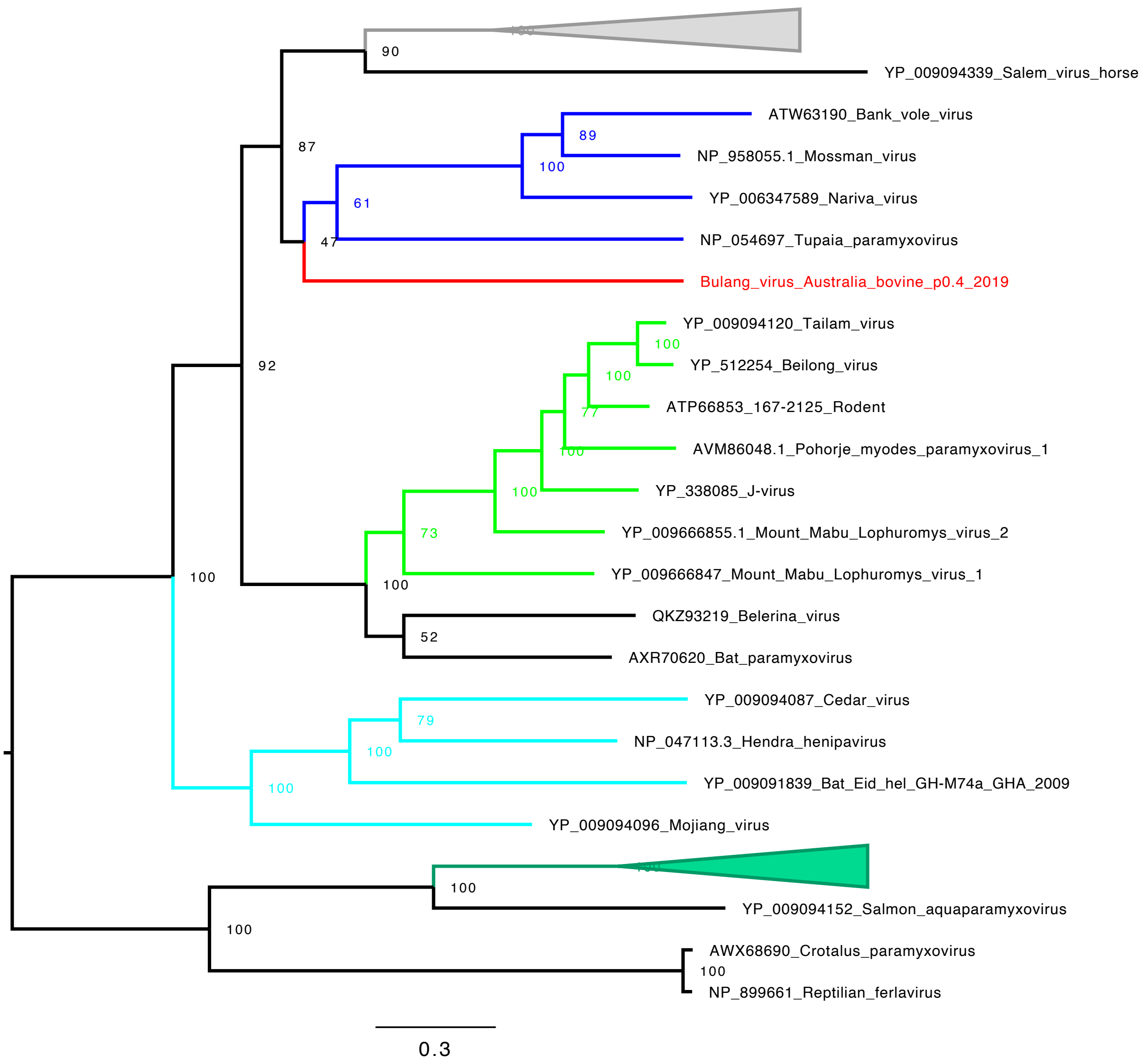

7.3. Bovine narmovirus-1 (Bulang virus)– Nucleoprotein

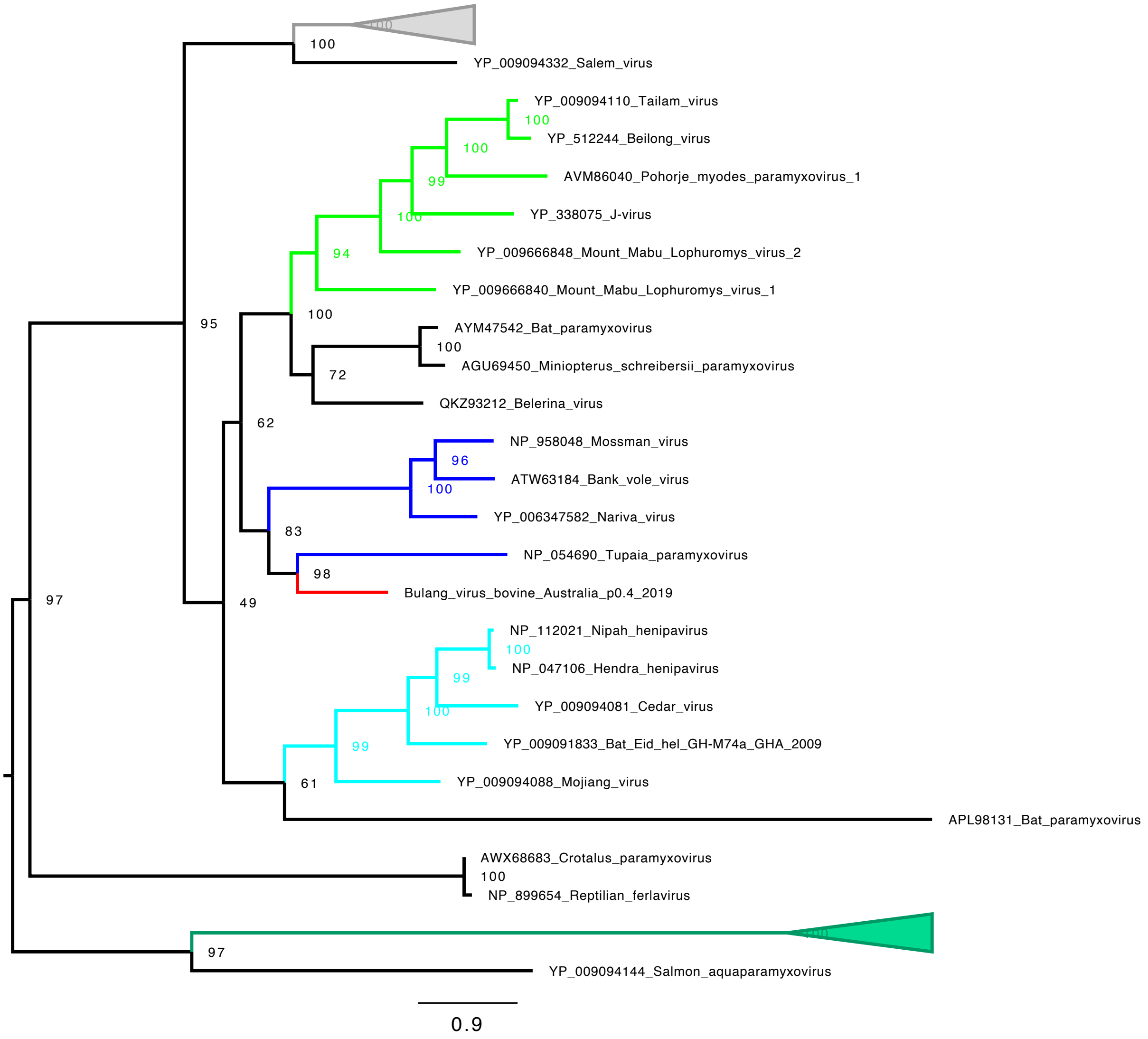

7.4. Bovine narmovirus-1 (Bulang virus)– Matrix

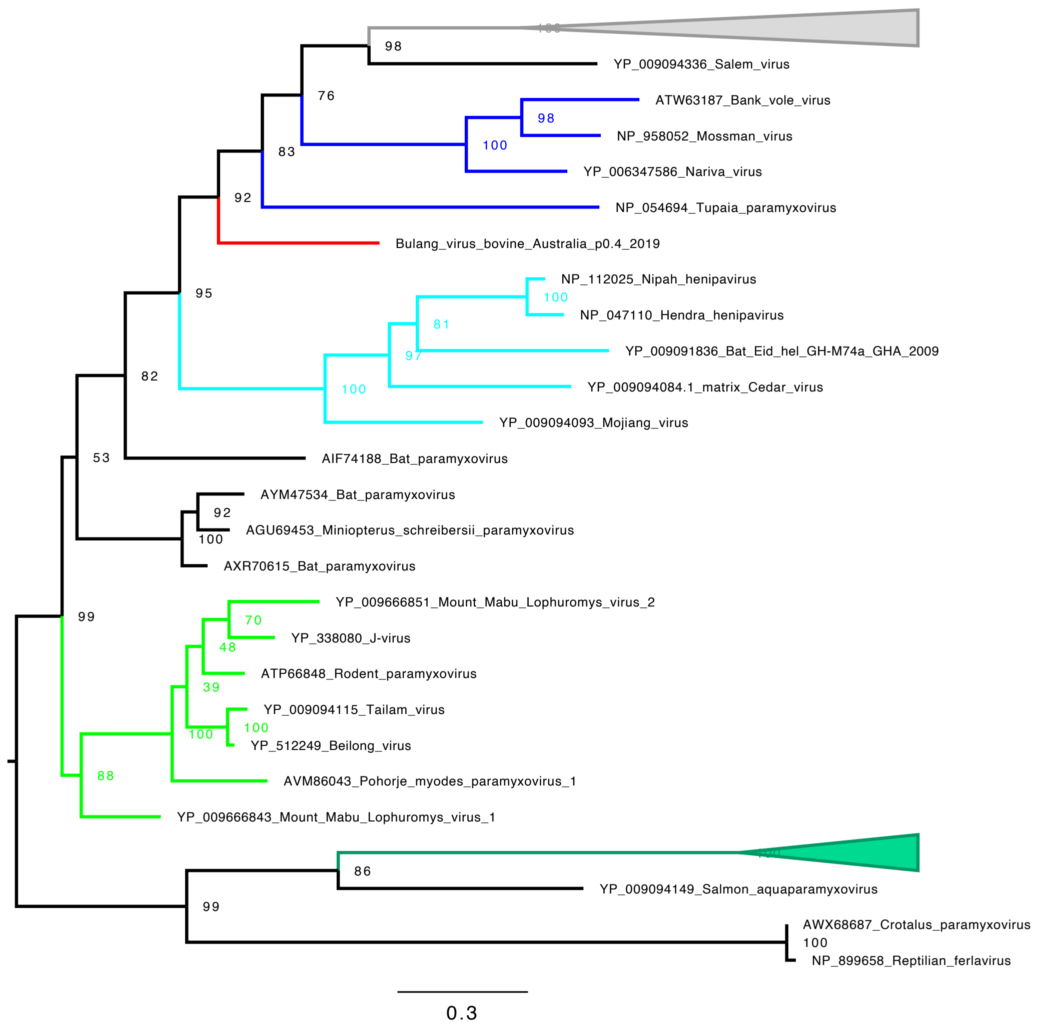

7.5. Bovine narmovirus-1 (Bulang virus)– Phosphoprotein

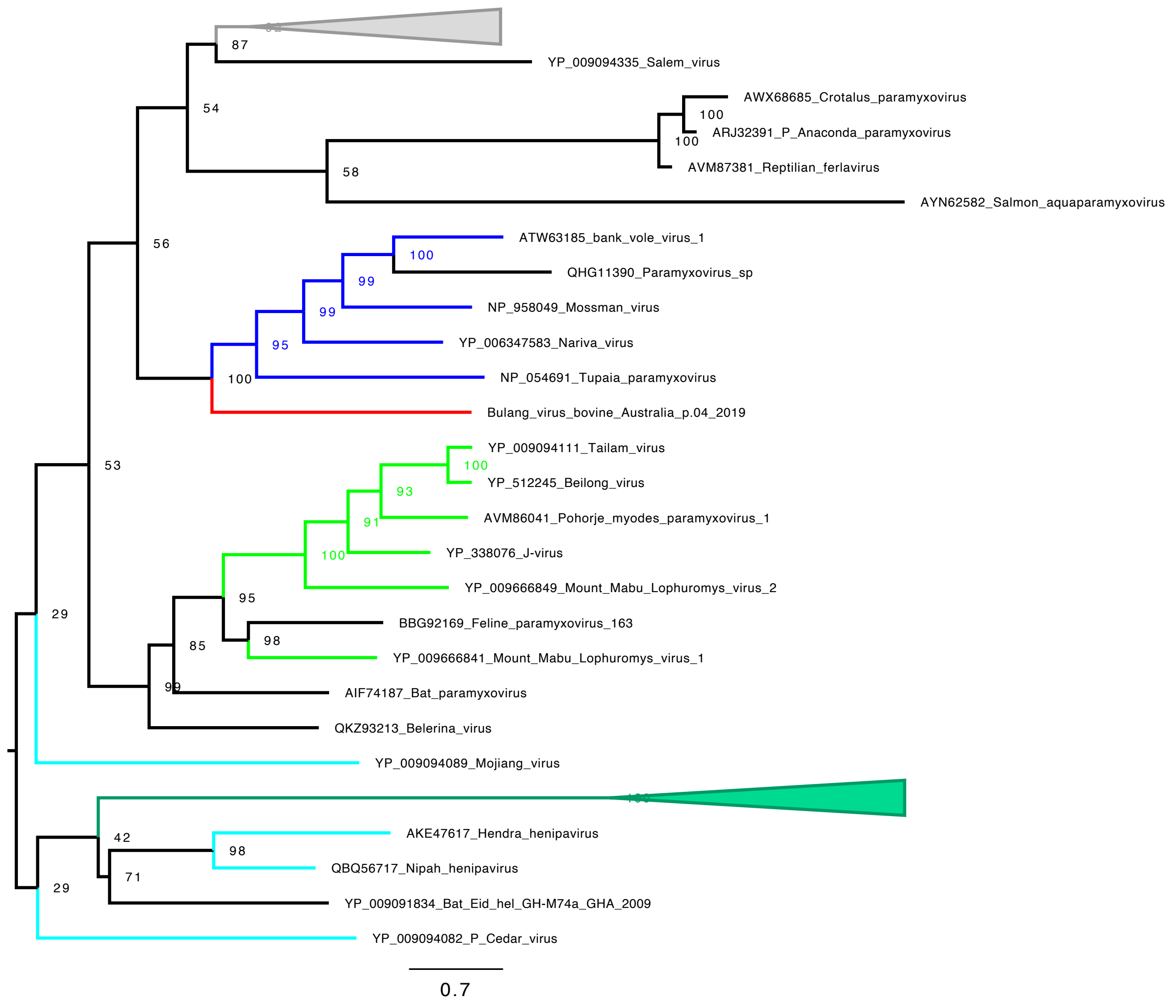

7.6. Bovine narmovirus-1 (Bulang virus)– Fusion protein

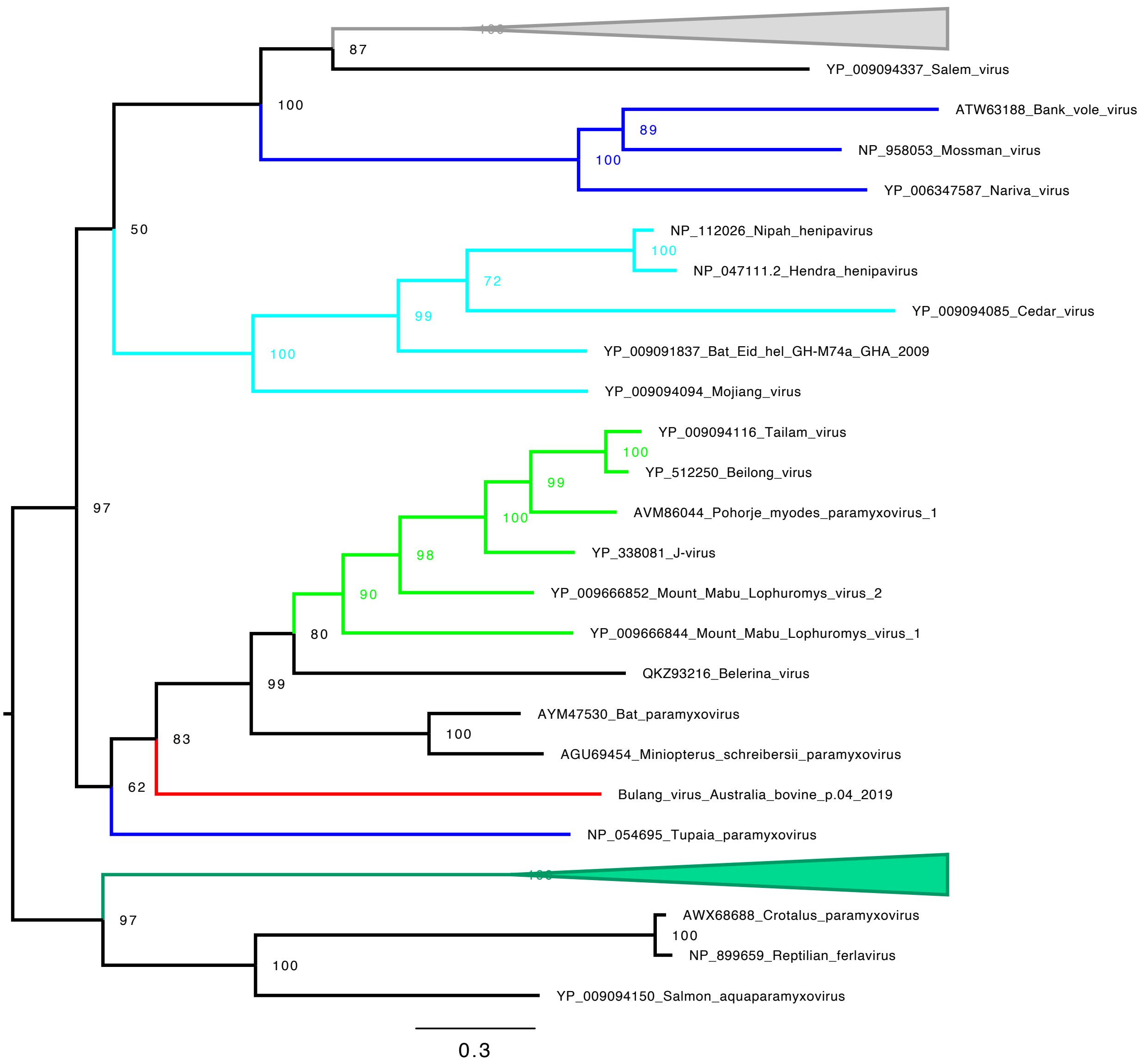

Supplement: Supplementary file 8 — Additional file 7. Overview of Bovine narmovirus-1 (Bulang virus) phylogenies. Fig 7.1. Maximum likelihood phylogenies of Bovine narmovirus-1 (Bulang virus). 7.2. Bovine narmovirus-1 (Bulang virus)– L protein. 7.3. Bovine narmovirus-1 (Bulang virus)– Nucleoprotein. 7.4. Bovine narmovirus-1 (Bulang virus)– Matrix. 7.5. Bovine narmovirus-1 (Bulang virus)– Phosphoprotein. 7.6. Bovine narmovirus-1 (Bulang virus)– Fusion protein. [file 40168_2023_1591_MOESM7_ESM.pdf]
