# Supplementary material for: Expanding the range of the respiratory infectome in Australian feedlot cattle with and without respiratory disease using metatranscriptomics
Source: Microbiome. 2023 Jul 25;11:158. doi: 10.1186/s40168-023-01591-1 (PMC10367309; doi:10.1186/s40168-023-01591-1)
Supplement: Supplementary file 9 — Additional file 8: Fig. Alpha and beta diversity of the metatranscriptomic sequence libraries obtained from animals with clinical bovine respiratory disease (cases) and without clinical disease (controls). Fig(a): The alpha diversity computed using different methods. Fig(b). NMDS plot using Bray-Curtis dissimilarities between samples in cases and controls. [file 40168_2023_1591_MOESM8_ESM.pdf]

Additional file 8

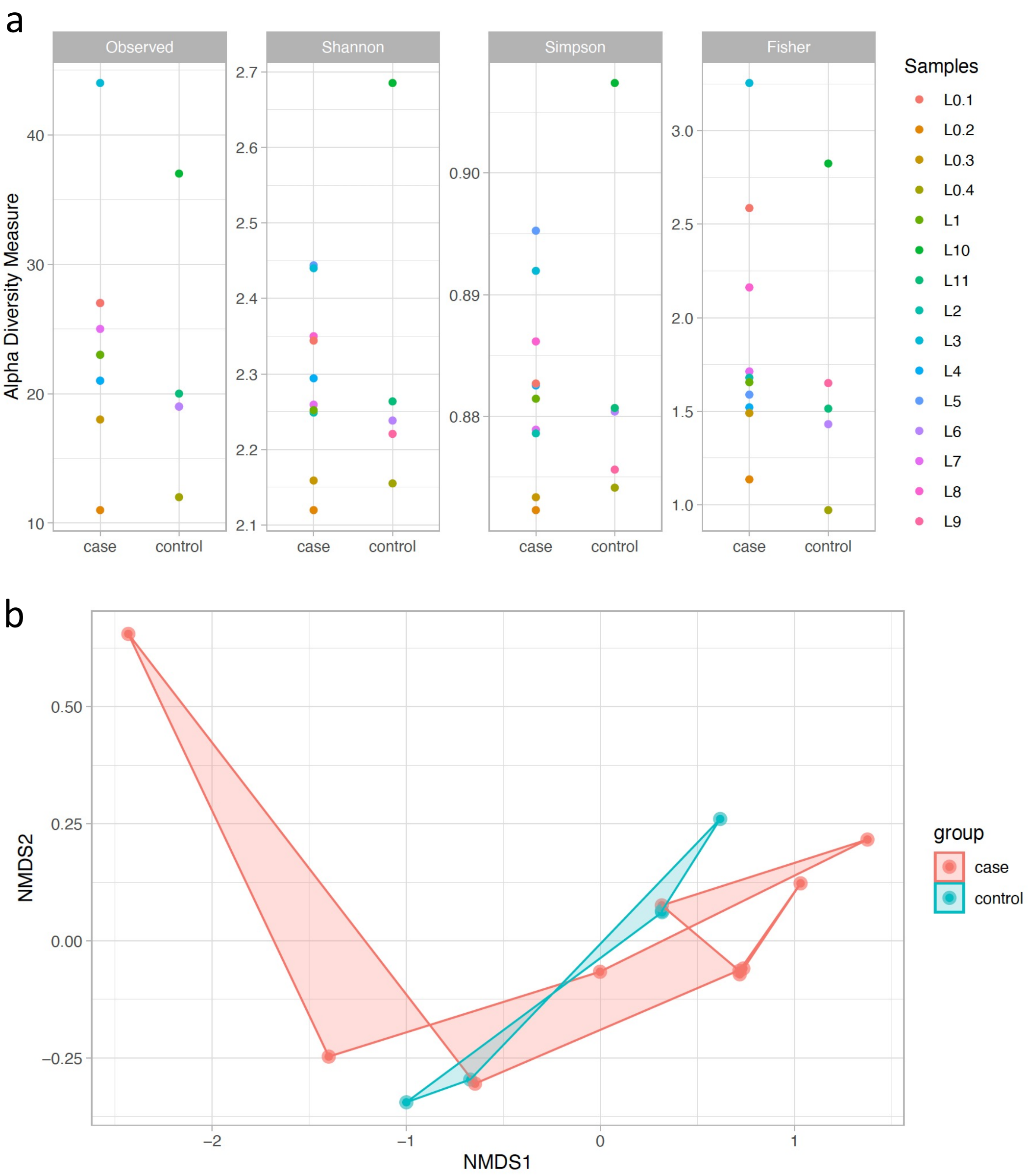

Fig: Alpha and beta diversity of the metatranscriptomic sequence libraries obtained from animals with clinical bovine respiratory disease (cases) and without clinical disease (controls). Fig(a): The alpha diversity computed using different methods, no significant differences in the alpha diversity were observed between cases and control. Fig(b). NMDS plot using Bray-Curtis dissimilarities between samples in cases and controls. There were no significant differences in the beta diversities between cases and controls.
